# Enhancing the Protein Stability of an Anticancer VHH‐Fc Heavy Chain Antibody through Computational Modeling and Variant Design

**DOI:** 10.1002/advs.202500004

**Published:** 2025-04-24

**Authors:** Yuan Fang, Menghua Song, Tianning Pu, Xiaoqing Song, Kailu Xu, Pengcheng Shen, Ting Cao, Yiman Zhao, Simon Hsu, Dongmei Han, Qiang Huang

**Affiliations:** ^1^ State Key Laboratory of Genetics and Development of Complex Phenotypes Shanghai Engineering Research Center of Industrial Microorganisms MOE Engineering Research Center of Gene Technology School of Life Sciences Fudan University Shanghai 200438 China; ^2^ Department of Technical Operations Shanghai Henlius Biotech, Inc. Shanghai 200233 China; ^3^ Multiscale Research Institute of Complex Systems Fudan University Shanghai 201203 China

**Keywords:** heavy chain antibody, protein design, single‐domain antibody, structure‐based modeling, therapeutic protein

## Abstract

VHHs (also known as nanobodies) are important therapeutic antibodies. To prolong their half‐life in bloodstream, VHHs are usually fused to the Fc fragment of full‐length antibodies. However, stability is often the main challenge for their commercialization, and methods to improve stability are still lacking. Here, an in silico pipeline is developed for analyzing the stability of an anticancer VHH‐Fc fusion antibody (VFA01) and designing its stable variants. Computational modeling is used to analyze the VFA01 structure and evaluate its conformational stability, disulfide bond reduction state, and aggregation and degradation tendency. By building mechanistic models of aggregation and degradation, the hotspot residues affecting stability: C130, F57, Y106, L120, and W111 are identified. Based on them, a series of VFA01 variants are designed and obtained a variant M11 (C130S/W111F/F57K) whose stability is significantly enhanced compared to VFA01: there are no visible particles in solution, and the change rate of DLS average hydrodynamic size, SEC HMW%, and CE‐SDS purity are improved by 6.2‐, 3.4‐, and 1.5‐fold, respectively. Both antigen‐binding activity and production yield are also improved by about 1.5‐fold. The results show that our computational pipeline is a very promising approach for improving the protein stability of therapeutic VHH‐Fc fusion antibodies.

## Graphical abstract

In this study, a computational pipeline is developed to systematically evaluate the conformational stability, disulfide bond reduction state, and aggregation and degradation tendencies of an anticancer VHH‐Fc fusion antibody. Based on the mechanistic insights, antibody variants with enhanced stability and increased yield are successfully designed. This study provides a promising approach for the optimization of therapeutic VHH‐Fc fusion antibodies.

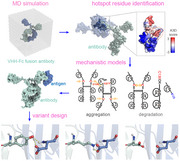

## 1 Introduction

In recent years, therapeutic proteins based on monoclonal antibodies have become a major part of the drug development pipelines of pharmaceutical companies. As of September 2024, >248 antibody therapies have been approved worldwide, including ≈188 approvals by the FDA (US Food and Drug Administration), 164 by the EMA (European Medicines Agency), and 84 by the NMPA (National Medical Products Administration of China), respectively, for the treatment of tumors, autoimmune disorders, ophthalmology, and several rare diseases, etc.^[^ ^1^ ^]^ However, the conventional IgG antibodies are relatively complicated in their structural formats (see also Figure S1A, Supporting Information), resulting in high development barriers and substantial production costs. Therefore, antibody miniaturization by genetic engineering has become an important trend in antibody research. In 1993, Hamers‐Casterman et al. discovered that camels have both conventional IgG1 antibodies with a molecular mass of ≈170 kDa, and heavy chain antibodies (HcAbs) without light chains and CH1 domains (see also Figure S1B, Supporting Information).^[^ ^2^ ^]^ Greenberg et al. subsequently found similar antibodies in other camelids, such as alpacas.^[^ ^3^ ^]^ HcAbs have a molecular weight of only 90 kDa. And their variable domain (known as VHH or nanobody) has a more compact structure that possesses a complete antigen‐binding sites and can specifically recognize hidden epitopes. In addition, VHHs are easily expressed and thus suitable for large‐scale commercial production. Because of these benefits, VHHs have become a new member of miniaturized antibodies, and are increasingly applied in disease diagnosis and treatment.^[^ ^4^, ^5^, ^6^, ^7^ ^]^

To improve the therapeutic efficacy of the native antibodies, researchers have used protein engineering to develop various antibody‐based protein therapeutics, such as VHH‐Fc fusion proteins, antibody‐drug conjugates (ADCs), bispecific antibodies, and multivalent/multi‐specific antibodies.^[^ ^8^, ^9^ ^]^ For example, VHH has been fused to the Fc fragment of human antibodies to prolong its half‐life in the blood via the neonatal Fc receptor (FcRn)‐mediated mechanism.^[^ ^10^ ^]^ Although the engineering improves the therapeutic efficacy of the antibodies, the physical and chemical stabilities resulting from the engineering remain a major challenge for their clinical application and commercialization. Physical stability includes conformational stability and colloidal stability: conformational stability implies the correct, functional folding of the protein structure, and colloidal stability indicates the good dispersion of the protein in a solution. Deficiencies in either conformational or colloidal stability can potentially increase protein‐protein interactions that trigger protein aggregation and thereby lead to protein particles or increase solution opalescence and viscosity of the protein therapeutics. Such problems can reduce their efficacy or production yield and even pose immunogenicity safety risks.^[^ ^11^, ^12^, ^13^ ^]^ As of March 2024, immunogenicity issues caused by particulate matter have been identified as the main reason for post‐marketing drug recalls.^[^ ^14^ ^]^ For example, Omontys (a pegylated peptide) was withdrawn due to its severe allergic reactions in 19 patients, including 2 deaths. And in February 2013, Takeda Pharmaceuticals and Affymax voluntarily recalled their drugs from the market.^[^ ^15^ ^]^ In March 2016, the FDA announced that high levels of subvisible particles were a potential cause of the severe allergic reactions associated with Omontys.^[^ ^15^ ^]^

Meanwhile, chemical stability involves the formation and/or cleavage of a certain number of covalent bonds of the engineered antibodies. These can alter the local hydrophobicity, charge distributions, and structures of the proteins, leading to reduced activity and immunogenicity. The main problems in the chemical stability are: oxidation of methionine or tryptophan, deamidation of asparagine residues, isomerization of aspartic acid, and the formation of succinimide intermediates.^[^ ^16^ ^]^ It is well known that in the discovery phase, the traditional screening methods focused primarily on the biological activity of the antibodies, and usually neglected the comprehensive assessment of their physicochemical properties. This oversight often led to insufficient stability and low production yields of the antibodies in the development phase, ultimately resulting in the failures of drug developments.^[^ ^11^, ^17^, ^18^ ^]^ However, there are limited reports on the stability assessment and optimization strategies for the engineered antibodies during the discovery phase. Therefore, understanding and addressing the stability issues of the therapeutic proteins is critical for their successful development.

**Figure 1:**
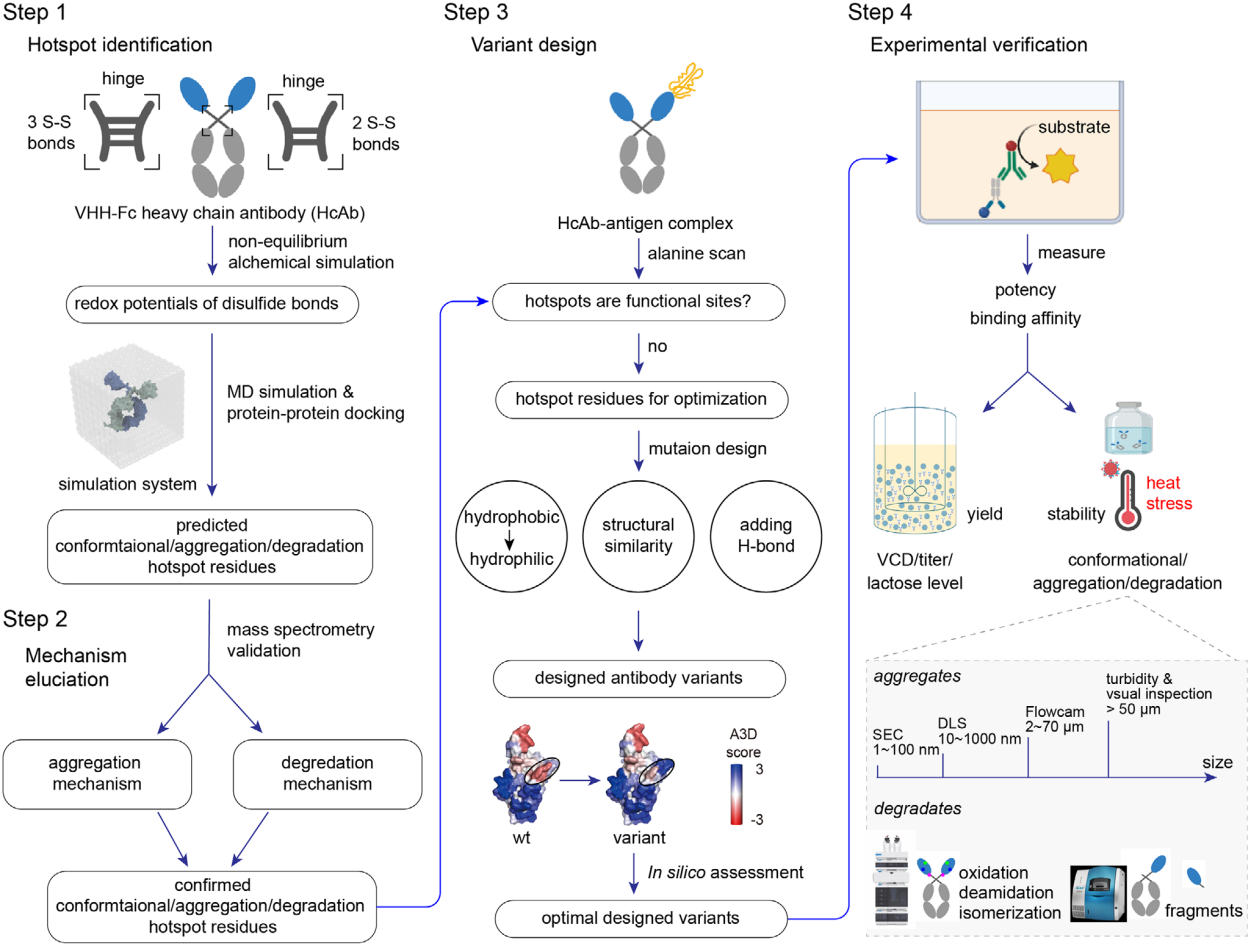
Workflow for stability assessment and variant design of the VHH‐Fc fusion antibody (HcAb).

In this study, we developed an in silico pipeline to assess the stability of an anticancer VHH‐Fc fusion antibody, named VFA01 and proposed a strategy to design stable variants (**Figure** **1**). VFA01 is an engineered antibody targeting the oncological antigen TIGIT,^[^ ^19^, ^20^ ^]^ which is currently in clinical trial. VFA01 consists of VHH, hinge, and Fc domains, forming a Y‐shaped structure with two identical heavy chains linked by three pairs of disulfide bonds in the hinge region via cysteine residues: C130, C136, and C139 (Figure S1B, Supporting Information). In the study, we used molecular dynamics (MD) simulations to analyze the dynamic structure of VFA01 and evaluated its conformational stability, disulfide bond reduction state, aggregation, and degradation tendency. By building the mechanistic models of aggregation and degradation, we identified the hotspot amino acids that affect the stability: C130, F57, Y106, L120, and W111. Based on these hotspot residues, we designed a series of VFA01 variants and validated them experimentally. Finally, we obtained an antibody variant M11 (C130S/W111F/F57K) whose stability was significantly improved compared to VFA01. Besides, both the antigen‐binding affinity and protein yield of this variant were also improved by about 1.5‐fold.

## 2 Results

### 2.1 Stability Profile of the VHH‐Fc Proteins

**Figure 2:**
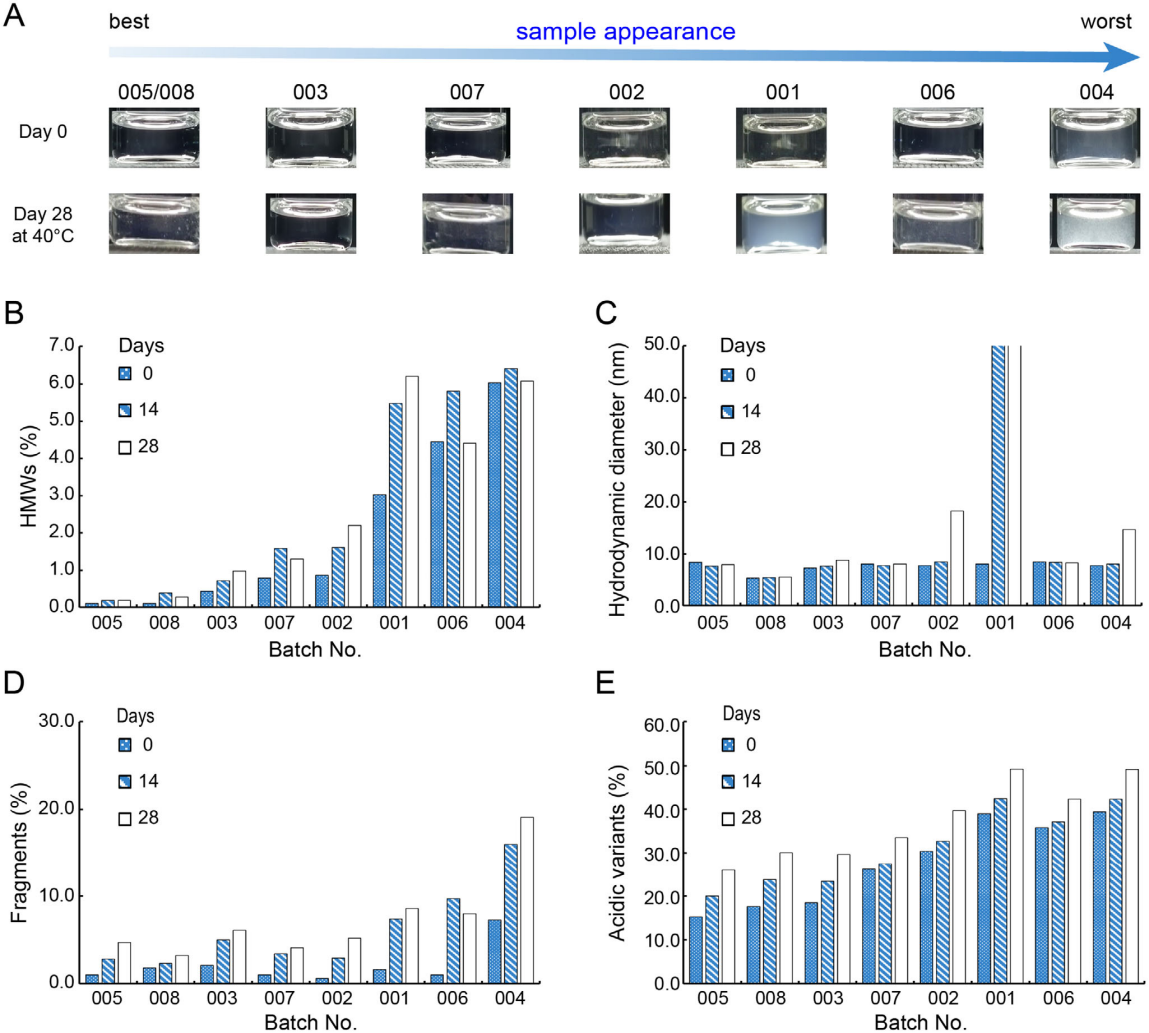
Protein stability comparison of VFA01 samples from 8 batches (Batch No. 001–008) of 5 L bioreactor production stressed at 40 °C for 4 weeks (28 days). A) Appearance. The arrow indicates the appearance of the samples from best to worst. B) HMW species (%) detected by SEC. C) Hydrodynamic diameter detected by DLS. D) Fragment species (%) detected by non‐reduced CE‐SDS. E) Acidic variants (%) detected by icIEF. Sampling points were set at day 0 (blue solid bars), day 14 (bars with blue diagonal slashes), and day 28 (colorless bars). In the figures, the batches are ranked in order of stability from best to worst. The measurements were performed under optimized conditions.

To evaluate the stability profile of VFA01, 8 batches of antibody proteins were produced in 5‐L bioreactors using different cell culture processes. The harvests of the cell culture fluids (HCCF) were purified by a 3‐step purification process and then formulated with the following components: 20 mg mL^−1^ VFA01, 20 mm histidine buffer at pH 6.0, 100 mm sucrose, and 0.02% polysorbate 20. The VFA01 samples obtained were filtrated, filled into glass vials, and stored at 40 °C for 4 weeks. The protein stability of the samples was then assessed by appearance, hydrodynamic diameter and polydisperse index measured by DLS (Dynamic Light Scattering), SEC‐HPLC (Size Exclusion Chromatography), icIEF (Imaged Capillary Isoelectric Focusing), and CE‐SDS (Capillary Electrophoresis Sodium Dodecyl Sulfate), respectively, and subvisible particles (SVP) by FlowCam at each sampling time (**Figure** **2**; Figure S2 of Supporting Information).

As shown in Figure 2, after 4 weeks of storage at 40 °C, the protein aggregates (size range from 10 nm to 100 µm), fragments, and acidic variants increased significantly in the formulation, indicating that the VFA01 proteins were undergoing aggregation and fragmentation. However, there was a distinct difference between the batches: batches 002, 003, 005, 007, and 008 remained essentially colorless and clear (Figure 2A); the SEC analysis showed a significant increase in HMW% for batches 001, 004, and 006, while only a slight increase in batches 002, 003, and 007 (Figure 2B); DLS showed an increase in the average hydrodynamic diameter for batches 001, 002, and 004 (Figure 2C), whereas batches 005 and 008 showed no significant changes in the SEC and DLS results. In contrast, batches 001, 006, and 004 exhibited more severe aggregation, appearing turbid with visible particles (Figure 2A), and batch 004 contained a greater amount of protein fragments (Figure 2D), batches 004, 001, 006, and 002 had a higher content of acidic variants (Figure 2E). Overall, the stability of the 8 batch samples of VFA01 under high‐temperature conditions is ranked from the best to the worst as follows: 005–008 > 003 > 007 > 002 > 001 > 006 > 004.

The above stability ranking showed a strong positive correlation with the attributes of the protein samples at the initial time (designated as T0): higher levels of acidic variants, aggregates, fragments, and non‐reducing heterogeneities at T0 resulted in poorer protein stability at the high temperature (Figure S2, Supporting Information). Obviously, the differences in the initial attributes could be attributed to their differences in the upstream production processes of the eight VFA01 batches. Therefore, early assessment and optimization of the protein stability of an engineered antibody is critical as it might shorten the development cycle of the production process and increase the success rate of drug development.

### 2.2 Hotspot Residues for Conformational Stability

**Figure 3:**
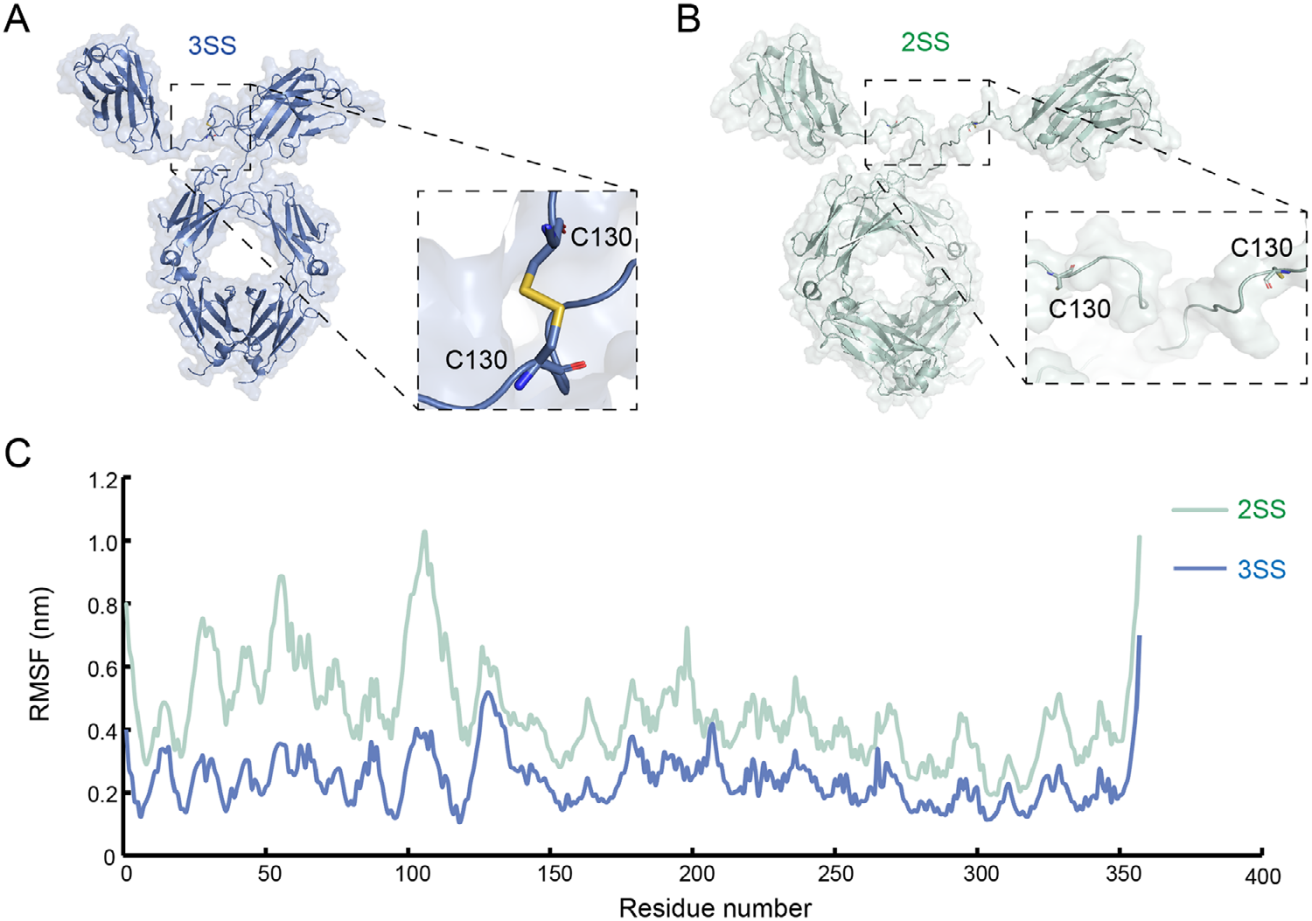
Representative structures of VFA01 3SS and 2SS and their structural fluctuations in the MD simulations. A) The structure of contains a hinge region with 3 disulfide bonds, where the C130 residues of two chains form a disulfide bond. B) The structure of 2SS with 2 disulfide bonds, where the C130 residues of two chains have no disulfide bond. C) The root mean square fluctuations (RMSFs) of 3SS and 2SS were calculated from the MD simulations.

To identify hotspot residues that affect the protein conformation, we first constructed an atomic model for the full‐length VFA01 structure using homology modeling and named it VFA01‐3SS (3SS for short) (**Figure** **3**A). We then used all‐atom molecular dynamics (MD) simulations to model its structural dynamics. The results of isothermal‐isobaric MD simulations showed that the RMSD (root mean square deviation) and Rg (radius of gyration) of the heavy backbone atoms of 3SS fluctuated slightly after 450 ns, indicating that the simulation system had reached the equilibrium state (Figure S3, Supporting Information). Therefore, we used the simulation trajectories after 450 ns for the subsequent conformational analysis.

Based on the simulation trajectories of 3SS, we calculated the redox potentials for the three interchain disulfide bonds (C130, C136, and C139) using the non‐equilibrium molecular simulations (NEMD) and evaluated their reduction propensities, because Lin et al. reported that when the redox potential of a disulfide bond is less than −330 mV, the disulfide bond can stabilize the protein structure; however, when the redox potential is high (−89 to −330 mV), the disulfide bond has the tendency to be reduced to free thiol groups (‐SH), which could lead to catalytic and allosteric effects.^[^ ^21^, ^22^, ^23^, ^24^ ^]^ The calculated redox potentials for the interchain disulfide bonds C130─C130, C136─C136, and C139─C139 in 3SS were −248.7, −434.8, and −448.6 mV, respectively (**Table** **1**). This indicated that the disulfide bond at C130 was susceptible to possible enzymatic reduction reactions during the cell culture and purification processes, resulting in the ‐SH groups. When in the presence of reactive oxygen species (ROS) in the solution, the ‐SH groups between two VFA01 proteins are likely to undergo oxidation reactions, forming intermolecular disulfide bonds and thus leading to oligomers.^[^ ^25^ ^]^

**Table 1:** Calculated reduction potentials of the 3 disulfide bonds in the hinge region.

Disulfide bond	ΔG [kJ mol^−1^]	Reduction potential (E_cal_) [mV]	Corrected reduction potential (E_corr_) E_corr_ = 1.5 E_ca l_ – 43 [mV]
Cys130‐Cys130	25.4	−137.1	−248.7
Cys136‐Cys136	48.3	−261.2	−434.8
Cys139‐Cys139	50.0	−270.4	−448.6

*Note*: The calculated data are presented as single values without SD.

To further elucidate the effect of the C130 disulfide bond on the conformational stability, we artificially reduced the C130─C130 disulfide bond and designed a variant protein with only two disulfide bonds in the hinge region (named VFA01‐2SS, 2SS for short). Subsequently, we performed 500 ns mD simulations on 2SS (Figure 3B), and then calculated the intramolecular free energy and hydrophobic patch area using the Rosetta and MOE software, and compared the results with those of 3SS (**Table** **2**). The average results were comparable between 3SS and 2SS in terms of pI values (9.0 vs 8.9), CamSol scores (−0.174 vs −0.180), and net charge (20.5 vs 21.3). However, the RMSFs of the hinge region in 2SS were larger than those in 3SS (Figure 3C), indicating that the structure of 2SS is more flexible. In addition, the intramolecular free energy (937.2 ± 120.4 kcal mol^−1^, *n* = 500) and the hydrophobic patch area (2391.1 ± 138.7 Å^2^, *n* = 500) of 2SS were significantly larger than those of 3SS (776.3 ± 115.1 kcal mol^−1^ and 2093.4 ± 131.6 Å^2^, *n* = 500, respectively), and the average percentage of solvent‐accessible surface area (SASA%) of C130 in 2SS reached as high as 58.8% (Table 2). These results suggest that 2SS has a lower conformational stability, and is more likely to undergo conformational changes, and thereby exposing its hydrophobic regions to the aqueous solvent, which is likely to trigger antibody aggregation mediated by hydrophobic interactions between two proteins.

**Table 2:** The calculated structural features of VFA01‐2SS and ‐3SS.

Variant	pI	SASA [%] of C130	Rosetta Δ*G* [kcal mol^−1^]	Hydrophobic patch area [Å^2^]	CamSol score	Net charge
2SS	8.9±0.1	58.8±9.3	937.2±120.4	2391.1±138.7	−0.180±0.014	21.3±0.6
3SS	9.0±0.1	36.0±8.4	776.3±115.3	2093.4±131.6	−0.174±0.013	20.5±0.4
*p*‐value	***	***	***	***	***	***

*Note*: The *p*‐value between two groups is determined by a paired sample *t*‐test (*** *p* < 0.0001).

To verify the computational results, we selected the samples from batches 008, 007, and 004 for mass spectrometry (MS) analysis. And their stability ranking from the best to the worst was 008 > 007 > 004. Based on the MS results, we then analyzed the correlation between the levels of the C130─C130 disulfide bond and the protein aggregation (**Table** **3**). As shown in Table 3, the levels of the C130─C130 disulfide bond in batches 008, 007, and 004 were 55.7%, 49.6%, and 35.5%, respectively, with the corresponding initial SEC HMW% levels of 0.1%, 0.6%, and 6.0%, respectively. This indicates that a higher presence of the C130─C130 disulfide bonds correlates with fewer initial protein aggregates and better stability performance, in agreement with the above computational analysis. Therefore, C130 of VFA01‐3SS is a hotspot residue of the conformational stability, and the reduction of the C130─C130 disulfide bond may expose the hydrophobic amino acids to the solvent, which likely promoted the formation of dimers or oligomers through the ‐SH or hydrophobic interactions.

**Table 3:** Correlation between the linkage state of C130 and the aggregation propensity in batches 004, 007, and 008.

Batch No.	C130 in free thiol state [%]	C130 in disulfide bond state [%]	SEC HMWs [%]	Rank of aggregation propensity at 40 °C
008	44.3	55.7	0.1	004 > 007 > 008
007	50.4	49.6	0.6	
004	64.5	35.5	6.0	

*Note*: The measurements were performed once under optimized conditions.

### 2.3 Hotspot Residues for Protein Aggregation

Besides C130, other residues could play a role in the VFA01 aggregation. To identify such hotspots, we next employed molecular docking to predict potential protein‐protein interaction (PPI) interface of VFA01 and combined it with the Aggrescan 3D (A3D) program to identify structural aggregation‐prone regions (STAP).^[^ ^26^, ^27^ ^]^ Given that VFA01 in solution may exist in the structural forms of 3SS and 2SS, we used the ClusPro software to perform docking simulations on the possible dimer combinations (3SS–3SS, 2SS–2SS, and 3SS–2SS), and a total of 254 low‐energy antibody dimer conformations were subsequently identified.^[^ ^28^ ^]^

**Figure 4:**
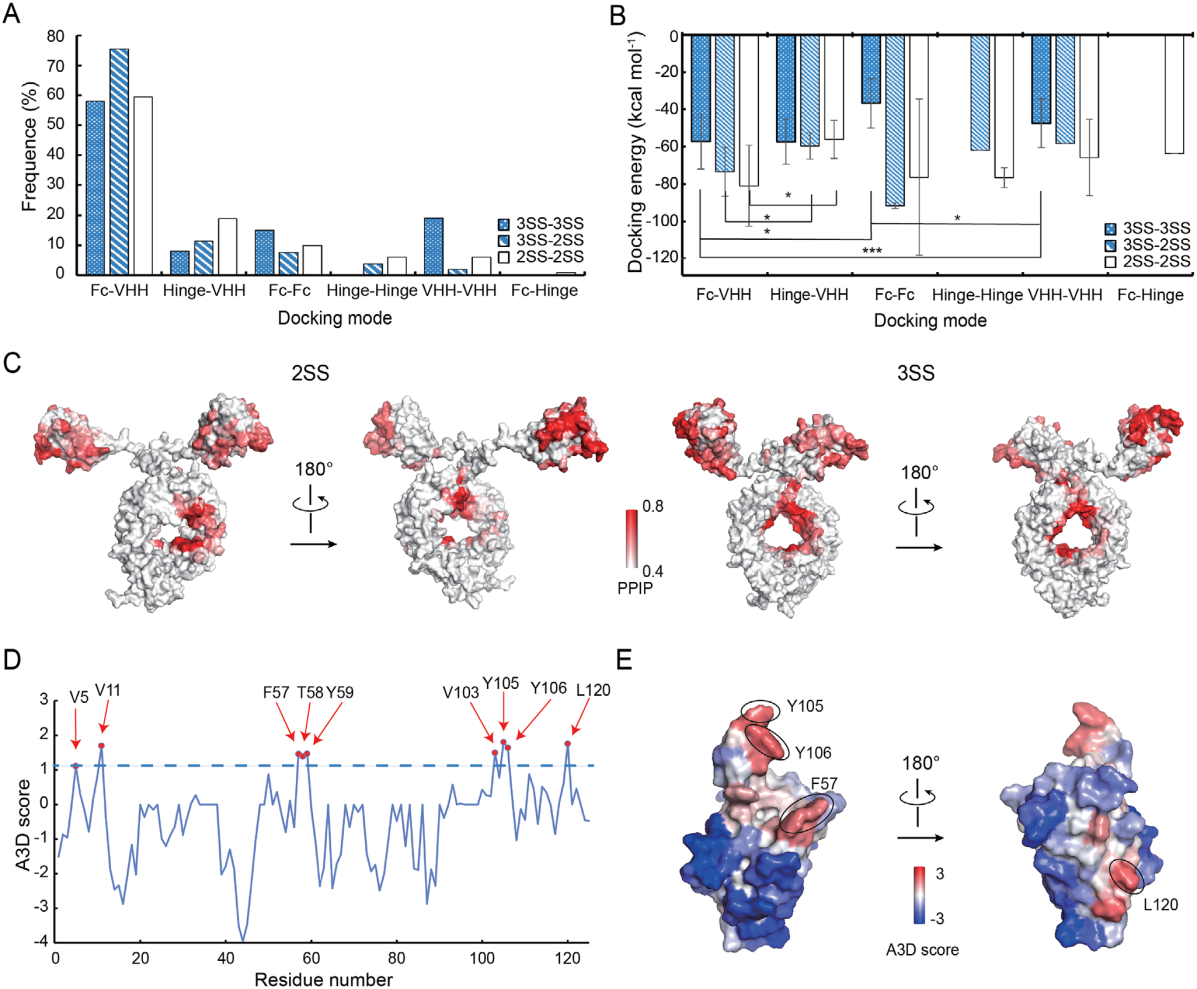
The PPI interfaces and aggregation hotspots of VFA01 identified by computational modeling. A) The percentages of six representative docking modes in the 3SS–3SS, 3SS–2SS, and 2SS–2SS docking simulations, respectively. B) Average docking energies of the six docking modes in the docking simulations. Data are presented as mean ± SD with the following sample sizes: *n*_3SS‐3SS_ = 58, n_2SS‐3SS_ = 40, *n*_2SS‐2SS_ = 61 for the Hinge‐VHH mode; *n*_2SS‐3SS_ = 6 and *n*_2SS‐2SS_ = 19 for the Hinge‐VHH mode; and *n*_3SS‐3SS_ = 19 for the VHH‐VHH mode. Sample sizes for other modes not mentioned above are zero. Statistical significance between groups was determined using a paired sample *t‐*test. Differences are considered significant at *p < 0.05, **p < 0.01, and ***p < 0.001, as indicated below the bars. C) Mapping of the PPI preference (PPIP) values onto the 2SS (left) and the 3SS (right) surfaces. The red areas are the aggregation hotspots. D) The A3D scores of the VHH domain, where those residues with a score value > 1 are marked in red. E) Mapping of the A3D scores onto the VHH structure, where the red areas are the positive peaks of the A3D curve and the blue areas are the negative dips, and the residues for mutations are indicated by their sequence numbers.

According to the structural domains where the binding interfaces are located, we classified the docking conformations into six different binding modes: Fc–Fc, Fc–VHH, Fc‐hinge, hinge‐hinge, hinge‐VHH, and VHH–VHH (Figure S4, Supporting Information). In the docking results of 3SS‐3SS, the majority of binding conformations were the Fc‐Fc (15.0%), Fc‐VHH (58.0%), and VHH–VHH (19.0%) modes, respectively (**Figure** **4**A). Among them, the average binding energy of Fc‐VHH was the lowest (−57.3 kcal mol^−1^) (Figure 4B), implying that Fc–VHH is the major binding mode for 3SS–3SS. We calculated the average PPI preference of each residue for this binding mode. Using a similar method, we also determined the PPI preference for each residue of 2SS–2SS and 2SS–3SS. By combining the three possible dimer combinations, we selected those residues with PPI preferences >0.5 to form the aggregation interface of VFA01 (Figure 4C).

Then, we used A3D to calculate the STAPs of the aggregation interfaces. Since Fc is a conserved domain of human antibodies that have been proven to have low stability risk, we focused on the STAPs of the VHH domain. A total of 9 amino acids exhibited an A3D score >1: V5, V11, F57, T58, Y59, V103, Y105, Y106, and L120 (Figure 4D). Among them, V5, V11, and V103 are not located on the aggregation interface. In addition, T58 and Y59 are conserved amino acids of VHH as identified by AbYsis (https://info.abysis.org/). By excluding these 5 residues, the STAP hotspots located at the aggregation interface are F57, Y105, Y106, and L120 (Figure 4E).

### 2.4 Hotspot Residues for Protein Oxidation

To identify the hotspot residues that trigger protein degradation, we analyzed potential oxidation, deamidation, and isomerization sites of VFA01. There are no obvious deamidation and isomerization sites in VFA01, so we focused on the oxidation sites. Protein oxidation typically occurs at methionine, cysteine, tryptophan, tyrosine, and histidine; and, methionine and tryptophan are the most susceptible.^[^ ^16^, ^29^ ^]^ Considering the residues with high oxidation risk are on the protein surface, we first calculated the SASA% of methionine and tryptophan on the VFA01 surface using the MD simulation trajectories.

**Figure 5:**
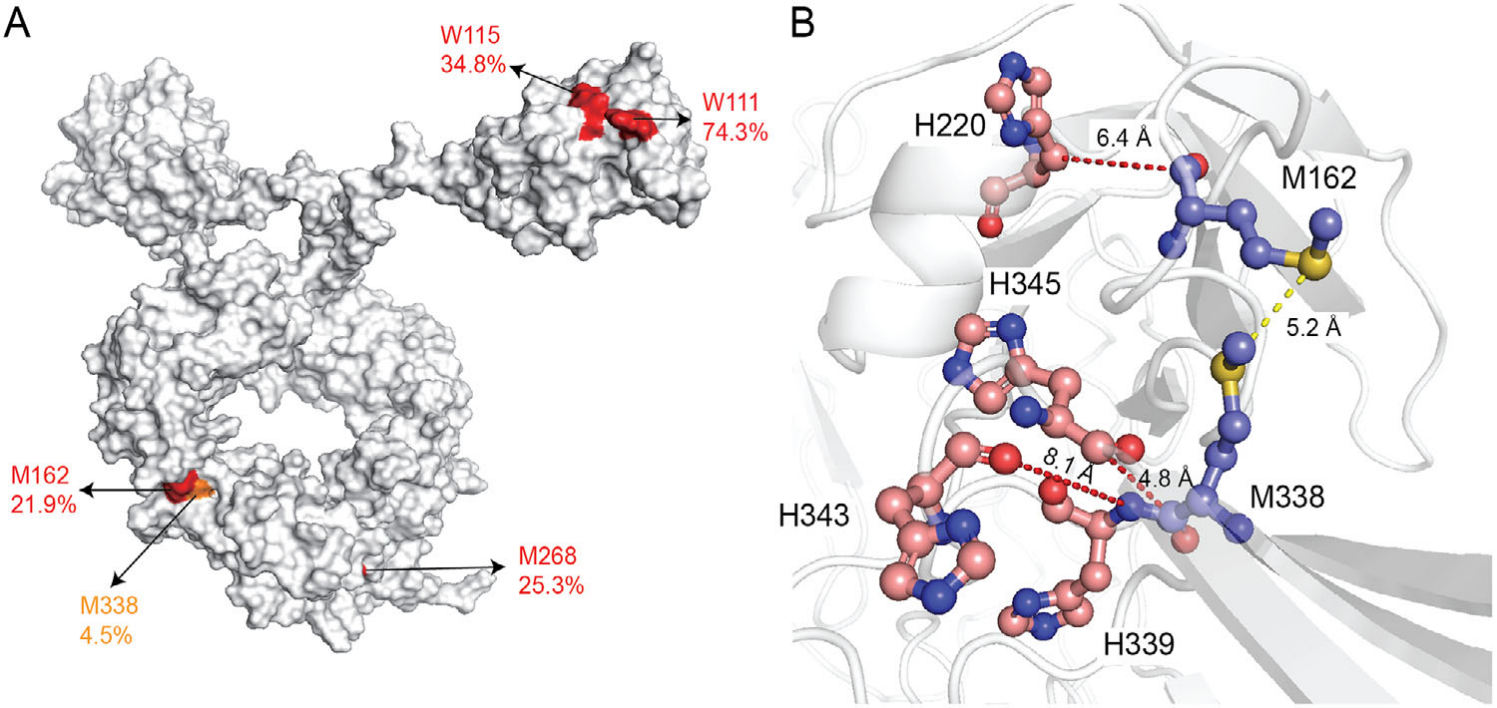
Computational prediction of the oxidation sites of VFA01. A) The Met (M) and Trp (W) sites that are highly susceptible to oxidation. The sites with SASA% > 20% are shown in red and those with SASA% < 20% are shown in orange. The SASA% values are given below the labels of the corresponding amino acids. B) The His (H) residues which are close to M162 and M338. The distance between the S atom of M162 and that of M338 is 5.2 Å, as indicated by the yellow dashed line. The red dashed lines indicate the closest distances of the surrounding His residues of Met. For M162, the closest is H220, with the closest distances of 6.4 Å. For M338, there are 3 surrounding His residues: H343, H345, and H339, with the closest distances being 8.1, 4.8, and 1.5 Å, respectively. Of these, H339 is the closest residue to M338, so the closest distance between them equals to ≈3.8 Å.

Sharma et al. have reported that methionine and tryptophan are more susceptible to oxidation reactions when their SASA% exceeds 20% and 30%, respectively.^[^ ^30^ ^]^ Our calculations showed that the SASA% values of W111, W115, M162, and M268 reached 73.4%, 34.8%, 21.9%, and 25.3%, respectively (**Figure** **5**A), suggesting a high risk of oxidation at these four sites. To confirm this, we performed mass spectrometry analysis on the samples from the 8 batches, and the results showed that the oxidation ratios at sites W111/W115, M338, and M162 were all larger than 0.5%, implying that they are highly susceptible to the oxidation. Moreover, the lower protein stability correlated with the higher oxidation ratio at these sites (**Table** **4**). However, M268, with a higher SASA% of 25.3%, exhibited no significant oxidation; in contrast, M338, with a lower SASA% of 4.5%, had some degree of oxidation. We speculated that the oxidation of M268 and M338 is not only related to their solvent exposure but also influenced by their neighboring residues.

**Table 4:** Correlation between the calculated solvent accessibility area and the oxidation level of the high‐risk residues (methionine and tryptophan).

Critical residue	SASA [Å^2^]	SASA [%]	Oxidation [%] of peptide mapping (ranked by stability from best to worst)							
			005	008	003	007	002	001	006	004	
M162	47.5	20.5	1.4	1.5	2.6	3.2	3.3	4.2	4.1	7.8	
M268	54.8	24.8	N.D.^a)^	N.D.	< 0.5	N.D.	< 0.5	0.5	0.5	0.7	
M338	9.80	1.50	0.7	1.1	0.9	1.0	1.3	1.4	1.4	2.5	
W111	200.1	69.2	0.5	1.2	2.7	2.8	2.7	4.9	4.9	7.7	
W115	93.9	28.0									

^a)^ N.D. – not detected. The measurements were performed once under optimized conditions.

As illustrated in Figure 5, M338 is adjacent to M162 with a SASA% of 21.9%, and the distance between their sulfur atoms is ≈5.2 Å (Figure 5A,B). And, within a radius of 10 Å around M338 and M162, there are four histidine residues: H220, H339, H345, and H343 (Figure 5B). Since the imidazole ring of histidine can catalyze the oxidation reaction of a neighboring methionine,^[^ ^31^ ^]^ M338 is likely to undergo oxidation under the catalysis of the M162 oxidation and surrounding histidine residues, making it a high‐risk oxidation site. For M268, we speculated that the reason for no oxidation is its different hydroxyl radicals from those of M162. The oxidation of methionine is typically induced by the hydroxyl radicals, and the unpaired electrons of hydroxyl radicals tend to attack groups with higher electron density.^[^ ^32^ ^]^ Considering that M268 is located at the Fc end of the heavy chain, we then used the electron density map of a similar protein with the same Fc end (PDB ID: 5VGP) as a structural reference. The reference map shows that its electron densities around M255 (corresponding to M162) and M431 (corresponding to M338) are normal, but those around M361 (corresponding to M268) are lower. It is likely that M268, despite the high solvent exposure rate, has a relatively low local electron density and is therefore less susceptible to the hydroxyl radical attack.

Taken together, the potential oxidation sites are W111, W115, M162, and M338. Of these, both M162 and M338 are located within the conserved Fc domain, and W115 has been identified as a conserved residue in the VHH framework. Since we have developed a standard platform production process that effectively modulates the oxidative states of these conserved residues, only W111, which is close to the CDR3 region, may pose an oxidation risk that could potentially affect the binding activity of VFA01, leading to stability issues in the drug formulation. Therefore, W111 is a key oxidation site that requires more attention.

### 2.5 Mechanistic Models for Aggregation and Degradation

**Figure 6:**
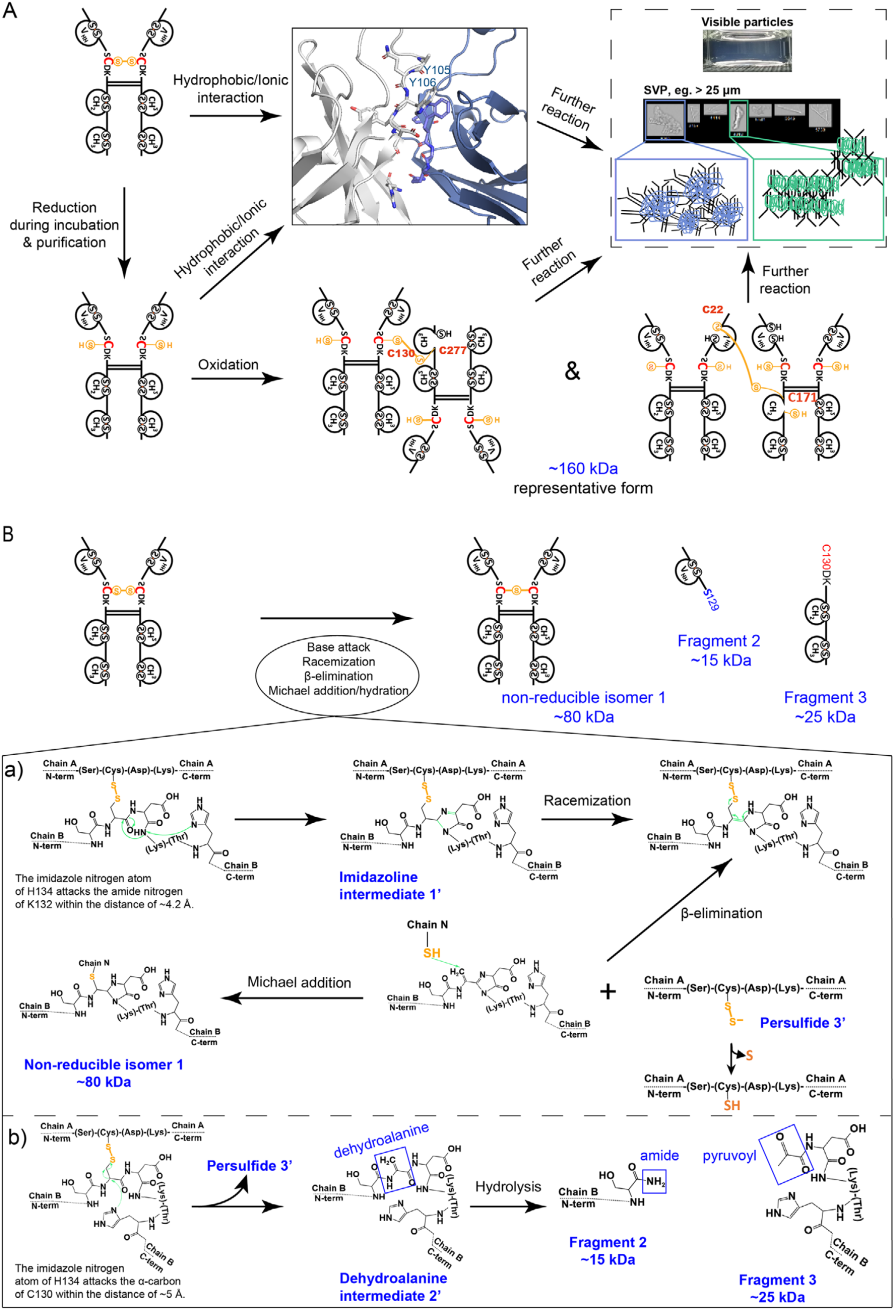
Schematic representation of the aggregation and degradation mechanisms of VFA01. A) The aggregation pathways. B) The degradation pathways, where a) is the pathway producing the non‐reducible isomer and b) is the pathway producing fragments 1 and 2. The reactions involving the cysteine residue (C130) are shown in red. The sulfur atoms and thiol groups in the cysteine residues are shown in yellow. The molecular weights of the aggregation and degradation products and their intermediates are shown in blue. The green arrows in the degradation pathways roughly indicate the reaction directions.

To confirm the identified hotspot residues, we constructed mechanistic models for the aggregation and degradation of VFA01. The above analysis has shown that C130 is a critical aggregation hotspot that tends to form intermolecular disulfide bonds. To verify this, we identified all the monomers and oligomers of VFA01 in the samples (Table S1, Supporting Information). We found that most of the oligomers can be reduced to monomers, indicating that the disulfide bonds are the main reason for the oligomer formation. As shown in Table S1, the content of C130─C277 in the oligomers was significantly higher than that in the monomers (20.3% vs 6.5%), and a slight difference was also found for C22─C171 (10.4% vs 7.8%). And the contents of other disulfide bonds were low, or their differences were small. Therefore, the oligomer formation is mainly attributed to the intermolecular disulfide bond of C130─C277. In addition, molecular docking results have indicated that VHH and the Fc domain could potentially form dimers or oligomers through hydrophobic interactions by F57, Y105, Y106, and L120. As a result, when the oligomers further aggregate, they could grow into particles with macroscopic length scales (>100 µm), increasing the opalescence effect, and ultimately producing insoluble particles or precipitation. Based on these analyses, a mechanistic model for the formation of the oligomers below 100 nm and the transition into visible protein particles has been illustrated in **Figure** **6**A.

Experimental analysis has also shown that VFA01 was degraded into fragments, non‐reducible isomers, and acidic isomers. Similarly, we used peptide mapping to characterize these fragments, and constructed a mechanistic model for the degradation, as shown in Figure 6B. The results showed that the non‐reducible isomers detected by R‐CE‐SDS were attributed to the formation of a thioether bond between SC^130^DK; a peptide bond cleavage between S129 and C130 was also found in the samples, generating a protein fragment with a molecular weight of ≈14 178 Da. We concluded that there were two fragmentation pathways: a) the nitrogen atom on the imidazole group of H134 attacked the amide nitrogen of Lys132, leading to a subsequent nucleophilic attack at the carbonyl carbon of C130 (distance < 4 Å), producing the imidazoline intermediate 1′, and the deprotonation of the alpha‐carbonyl carbon keto‐enol tautomer led to the conjugation of the imidazole ring, promoting the racemization of C130; and the transfer of the carbon–carbon double bond to the alpha, beta‐unsaturated dehydroalanine residue triggered the beta‐elimination of the disulfide bond, resulting in persulfide 3′. Since persulfide 3′ has the oxidizing ability, it may increase the oxidation ratio of W111/W115, M162, and M338, and then lead to the acidic variants; b) if the nitrogen atom on the imidazole group of H134 attacked the carbon atom next to the alpha‐carbonyl carbon, a dehydroalanine intermediate 2′ was produced. The intermediate subsequently underwent a hydrolysis reaction at the N‐terminal site of the dehydroalanine group, resulting in the cleavage of the peptide bond between S129 and C130. This process produced a noncovalent amide at the VHH end (Fragment 2, with a molecular weight of ≈15 kDa) and a pyruvoyl group at the Fc end (Fragment 3, with a molecular weight of ≈25 kDa).^[^ ^33^, ^34^, ^35^ ^]^

### 2.6 Design of VHH‐Fc Antibody Variants

To improve the protein stability, we designed VFA01 variants by introducing amino acid mutations at the identified hotspots: C130, F57, Y105, Y106, L120, and W111, according to the following rationales: 1) avoiding disruption of the antibody‐antigen binding by preserving the key antibody‐antigen interactions; 2) replacing the hydrophobic amino acids with the hydrophilic/charged amino acids (such as lysine K or aspartate D) to reduce the propensity for protein aggregation by hydrophobic attraction and increase their electrostatic repulsion; 3) optimizing the oxidation‐prone sites by changing W111 to phenylalanine F, which has similar side chain without the risk of oxidation; 4) mutating C130 to a serine S residue with a chemically similar ‐OH group to eliminate the issues caused by the reduction of C130.

**Figure 7:**
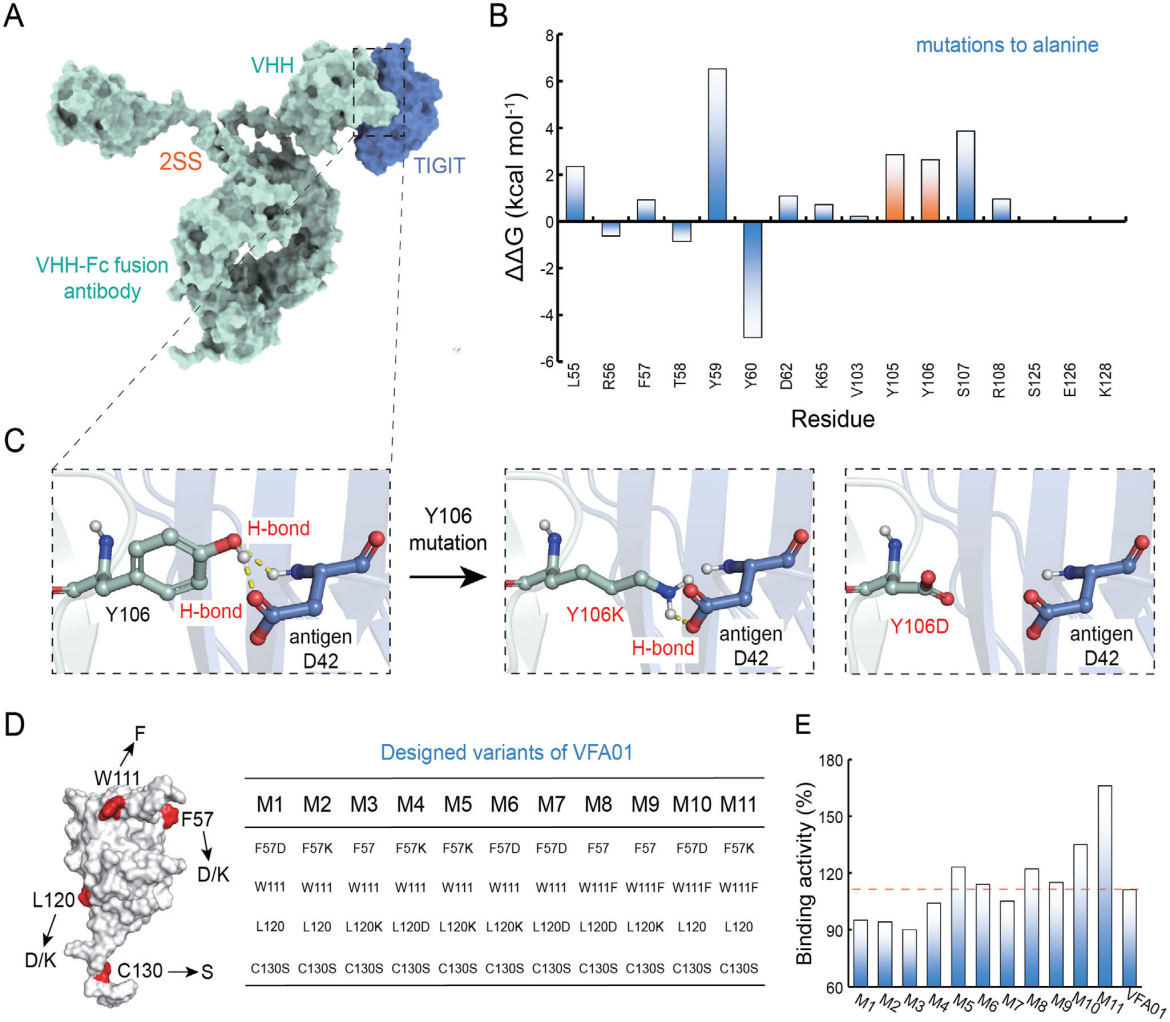
Rational design of VFA01 variants. A) The complex structure of 2SS (green) and antigen (blue). B) The ΔΔG binding changes upon alanine mutation for the 2SS‐antigen complex. Residues Y105 and Y106 at the aggregation interface are shown in orange, and others shown in blue. C) The H‐bond changes upon mutation of Y106 to K106 or D106. The H‐bond is shown as yellow dashed lines. D) The residues for the point mutations (red). Mutation combinations form the variants M1–M11, as shown in the right panel. E) The experimental binding activities of VFA01 and its variants. The measurements were performed once under optimized conditions.

Molecular docking was first used to assess the potential impact of the hotspots on the antibody‐antigen binding affinity. We utilized the ClusPro software for the protein−protein docking between 2SS and the antigen TIGIT, and obtained several complexes with different conformations. We then selected the conformations with the lowest binding energy for alanine scanning calculations (**Figure** **7**A). The results showed that when L55, Y59, D62, Y105, Y106, and S107 were mutated to alanine, the change in the free energy (ΔΔG) between the antibody and antigen was greater than 1.0 kcal mol^−1^, suggesting that the mutations at these sites could potentially weaken the antibody‐antigen interactions (Figure 7B). To further confirm the predictions, we mutated Y106 to D or K to examine the effect of the mutation on binding affinity. In the 2SS‐TIGIT complex, Y106 forms two hydrogen bonds with TIGIT; when Y106 was mutated to D or K, the number of the hydrogen bonds decreased, suggesting that the binding activity might be reduced by the mutation (Figure 7C). Indeed, experiments confirmed that the mutation of Y106 to D or K completely abolished the binding activity. Therefore, mutations of Y105 and Y106 were not considered.

According to the above rationales and analyses, we designed a series of VFA01 variants by combining amino acid mutations as follows: C130 was mutated to S, L120 and F57 were mutated to either D or K, and W111 was mutated to F. Thus, a total of 11 variants were designed and validated by experiments (Figure 7D). The antigen binding affinity assays showed that compared to VFA01, all the designed variants retained more than 90% binding activities. Notably, one of the variants, M11 (C130S/W111F/F57K), showed improved antigen binding capability, with a 1.5‐fold increase in the binding activity (Figure 7E).

### 2.7 Protein Stability and Production Yield of Designed Variants

**Figure 8:**
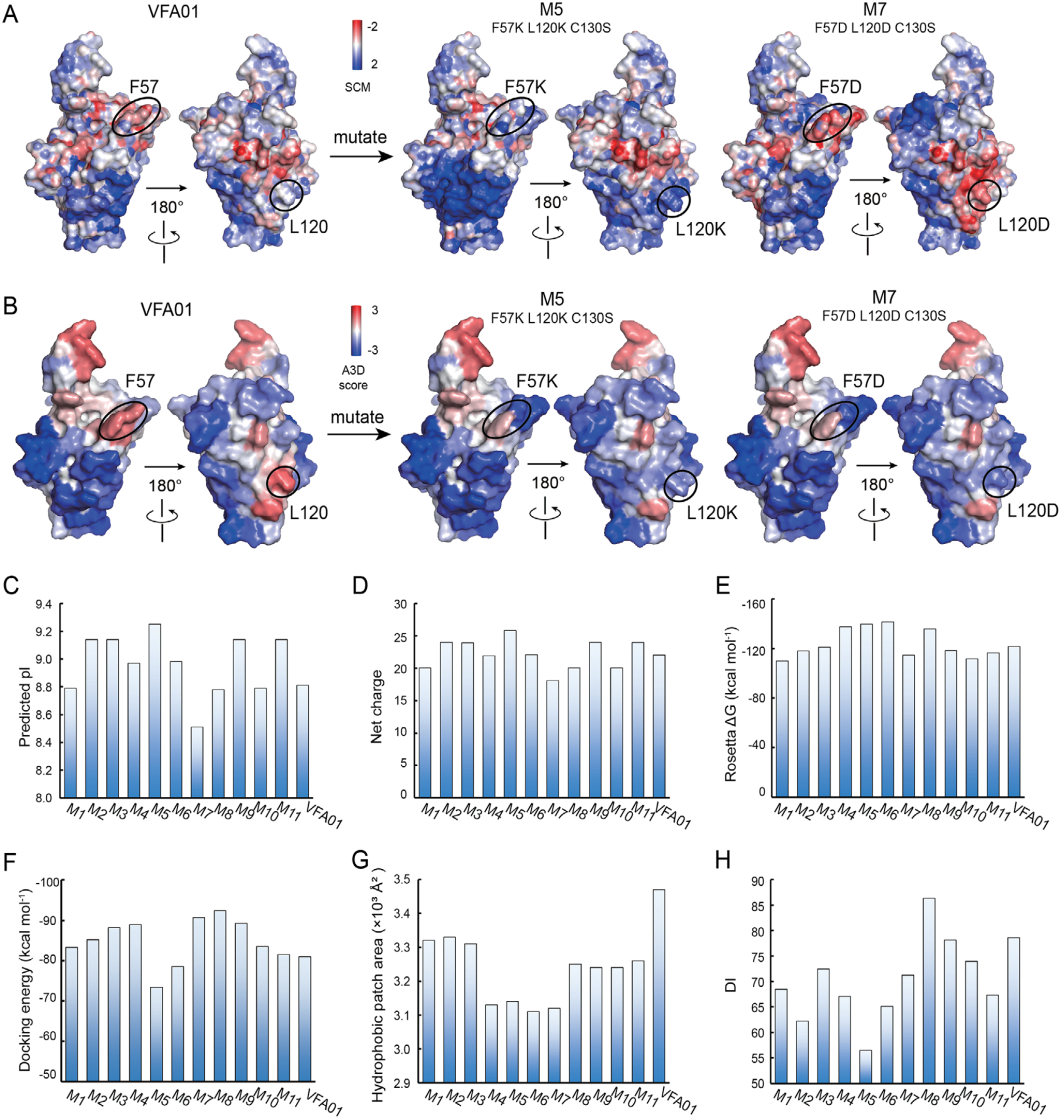
Computational evaluation of VFA01 and its variants. A) The SCM (spatial charge map) of VFA01 and its variants M5 and M7, showing how the mutations affect the electrostatic patch. The SCM changes caused by the F57 and L120 mutations are highlighted by the black circle. Red patches indicate the electronegative regions, while blue patches indicate the electropositive patches. B) The A3D scores of VFA01, M5, and M7, showing how the mutations affect hydrophobicity. The A3D changes caused by the F57 and L120 mutations are highlighted by the black circle. Red patches indicate the hydrophobic regions, while blue patches indicate the hydrophilic regions. C) Predicted pI values. D) Predicted net charge values. E) Rosetta energies. F) The average docking energies of the main Fc‐VHH docking mode. G) Hydrophobic patch areas. H) DI (Developability Index) values. All the calculated data were derived from the static 3D structures and thus are presented as single values without SD.

**Figure 9:**
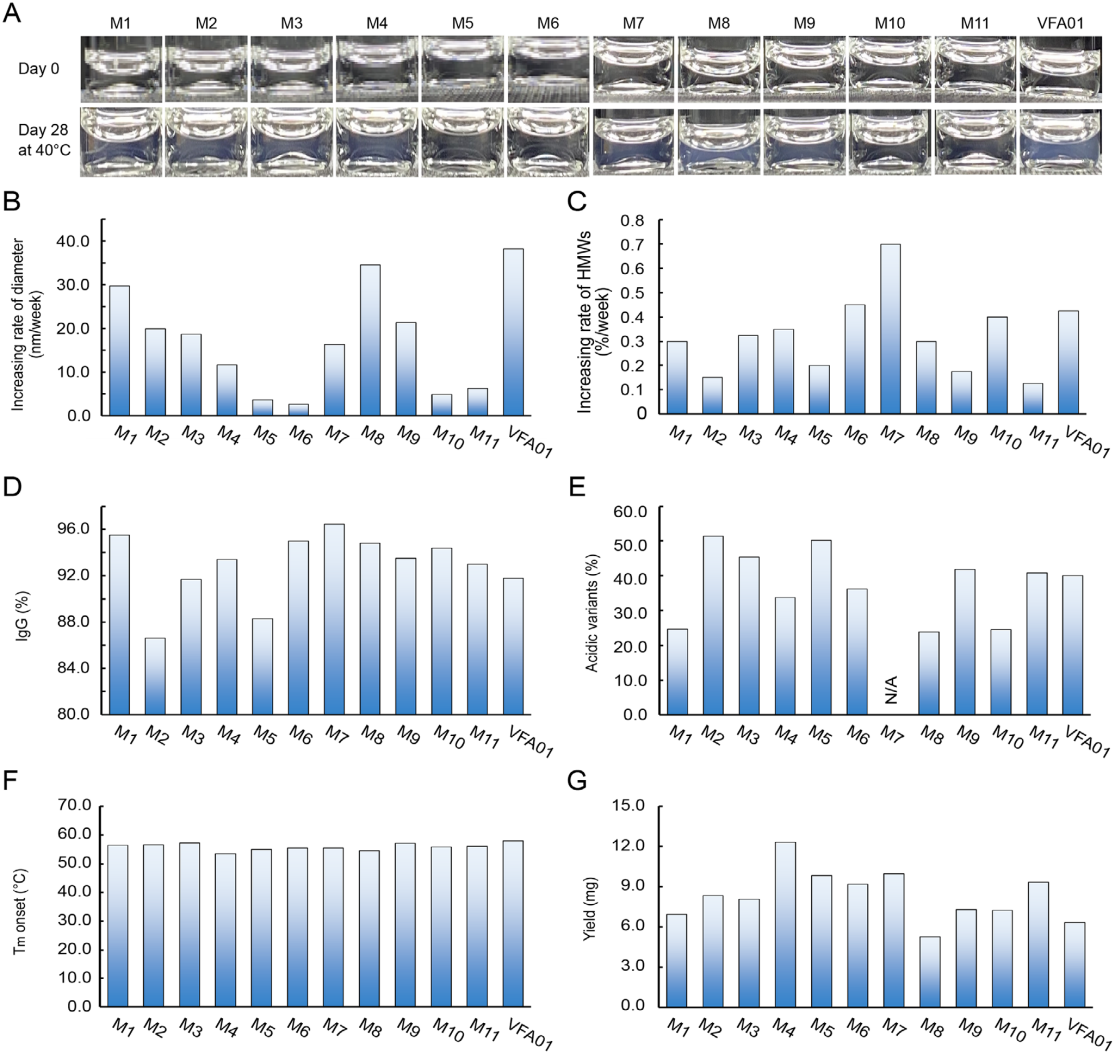
Experimental results of VFA01 and its variants stressed at 40 °C for 4 weeks. A) Appearance. B) Rate of diameter increase detected by DLS. C) Rate of increase of high molecular weight (HMW) species detected by SEC. D) IgG detected by non‐reduced CE‐SDS at the initial timepoint. E) Acidic variants detected by icIEF at the initial timepoint. Note that the pI of M7 dropped to 7.8 and caused the peaks to merge together, so the ratio of acidic variants could not be acquired. F) Tm onset detected by DSF. G) Yield. The measurements were performed once under optimized conditions.

To computationally evaluate the stability of the designed variants, we built their full‐length models according to the workflow in Figure 1, and then calculated their isoelectric point (pI) values, conformational stability, and aggregation tendencies, respectively. As shown in **Figure** **8**, mutation of F57 and/or L120 to the positively charged K resulted in an increased pI and a higher net charge. In contrast, mutation to the negatively charged D decreased the pI value and reduced the net charge. In particular, for variant M7 (C130S/F57D/L120D), with two D mutations, the theoretical pI decreased to 8.5 (Figure 8A,C,D). This suggests that simultaneous mutations of multiple sites to D altered the charge distribution of the protein and significantly lowered the pI value and the net charge, thereby reducing to the protein colloidal stability. DSF profiles also revealed that M4 (C130S/F57K/L120D), M7 (C130S/F57D/L120D), and M8 (C130S/W111F/L120D) showed significant differences from VFA01 (Figure S5, Supporting Information), suggesting an aggregation occurring prior to the protein unfolding. Since all three variants contain L120D, we speculated that the negatively charged D enhanced the colloidal interactions between the proteins, leading to the aggregation. Meanwhile, we calculated the folding free energies for the variants and VFA01 using the Rosetta software, and found their ΔG values in the range from −110 to −140 kcal mol^−1^, indicating that the introduced mutations did not significantly affect the conformational stability of the variants (Figure 8E). Consistent with the computational analysis, the DSF data also showed no significant difference in T_m onset_ between the variants and VFA01 (**Figure** **9**F; Figure S5 in Supporting Information). Taken together, the negatively charged D may not significantly affect the protein conformation but could result in poor colloidal stability, and thus promoting the aggregation.

Next, we evaluated the aggregation tendency of the variants by calculating the docking energy, hydrophobic patch area, and developability index (DI), respectively. Docking energy calculations showed that M5 (C130S/F57K/L120K), M6 (C130S/F57D/L120K) displayed a reduced aggregation compared to VFA01 and the other variants (Figure 8F). Hydrophobic patch area calculations showed that the hydrophobic patch area of each variant is smaller than that of VFA01, implying a reduced risk of aggregation through hydrophobic interactions. Meanwhile, M4‐M7 showed smaller hydrophobic patch area among the variants (Figure 8B,G). Computational DI values, which combine hydrophobic and electrostatic effects, showed that except for M8, the values of the other variants are lower than that of VFA01, indicating increased stability. Among them, M5 has the lowest DI value (Figure 8H). To confirm these, we performed an accelerated test at 40 °C for the variants, and the results are shown in Figure 9. As shown, after 4 weeks at 40 °C, VFA01 had more heavy opalescent appearance with obvious visible particles; in contrast, M5 (C130S/F57K/L120K), M6 (C130S/F57D/L120K), M10 (C130S/F57D/W111F), and M11 (C130S/F57K/W111F) had a clearer appearance (Figure 9A), and their DLS average hydrodynamic size growth rates were 3.6, 2.6, 4.9, and 6.2 nm per week, respectively, which were also significantly lower than that of VFA01 (38.2 nm per week) (Figure 9B). This indicates that the introduced mutations effectively inhibited the protein aggregation in their samples. However, M7 and M8 were slightly less effective in inhibiting the aggregation because M8 had the fastest DLS hydrodynamic size growth rate (34.5 nm per week), and M7 had the fastest SEC HMW% increment rate (0.7% per week), which is higher than that of VFA01 (0.4% per week) (Figure 9B,C). Overall, it can be concluded that the mutation to K can inhibit the aggregation, but the mutation to D plays an opposite role.

We also investigated the possible fragments and oxidation species using CE‐SDS and peptide mapping (Figure S6, Supporting Information). We found that all the variants mutating C130 to S significantly thwarted the breakage of the disulfide bond at SC^130^DK (Figure S6A, Supporting Information); meanwhile, the variants M8‐M11 mutating W111 to F addressed the oxidation issue (Figure S6B, Supporting Information). On the contrary, compared to VFA01, M2 (C130S/F57K) and M5 (C130S/F57K/L120K) produced more fragments at the initial time (T0), indicating that these two variants underwent more peptide bond breakage during the cell culture and purification processes (Figure 9D). At the same time, icIEF results also showed that the acidic species of M2 and M5 were significantly more than those of VFA01 (Figure 9E). This suggested that the mutation to K accelerated the protein degradation, resulting in the fragments and the acidic species.

Furthermore, we examined the production yield of each variant, in order to assess the impact of the mutations on their production. Notably, M4 (C130S/F57K/L120D), M5 (C130S/F57K/L120K), M6 (C130S/F57D/L120K), M7 (C130S/F57D/L120D), and M11 (C130S/W111F/F57D) showed improved production yields, with 1.5–2.0‐fold increment compared to that of VFA01. And, M8 (C130S/W111F/L120D) exhibited the lowest yield (Figure 9G). Significantly, the variants with lower aggregation, such as M5, M6, and M11, also showed increased yields, indicating a strong correlation between the low protein aggregation tendency and the high yield. So, guided by the computational analyses and evaluations, we have successfully designed VFA01 variants (M6 and M11) that improved the protein stability and increased the production yield.

### 2.8 Correlations between Calculated Structural Features and Antibody Stability

**Figure 10:**
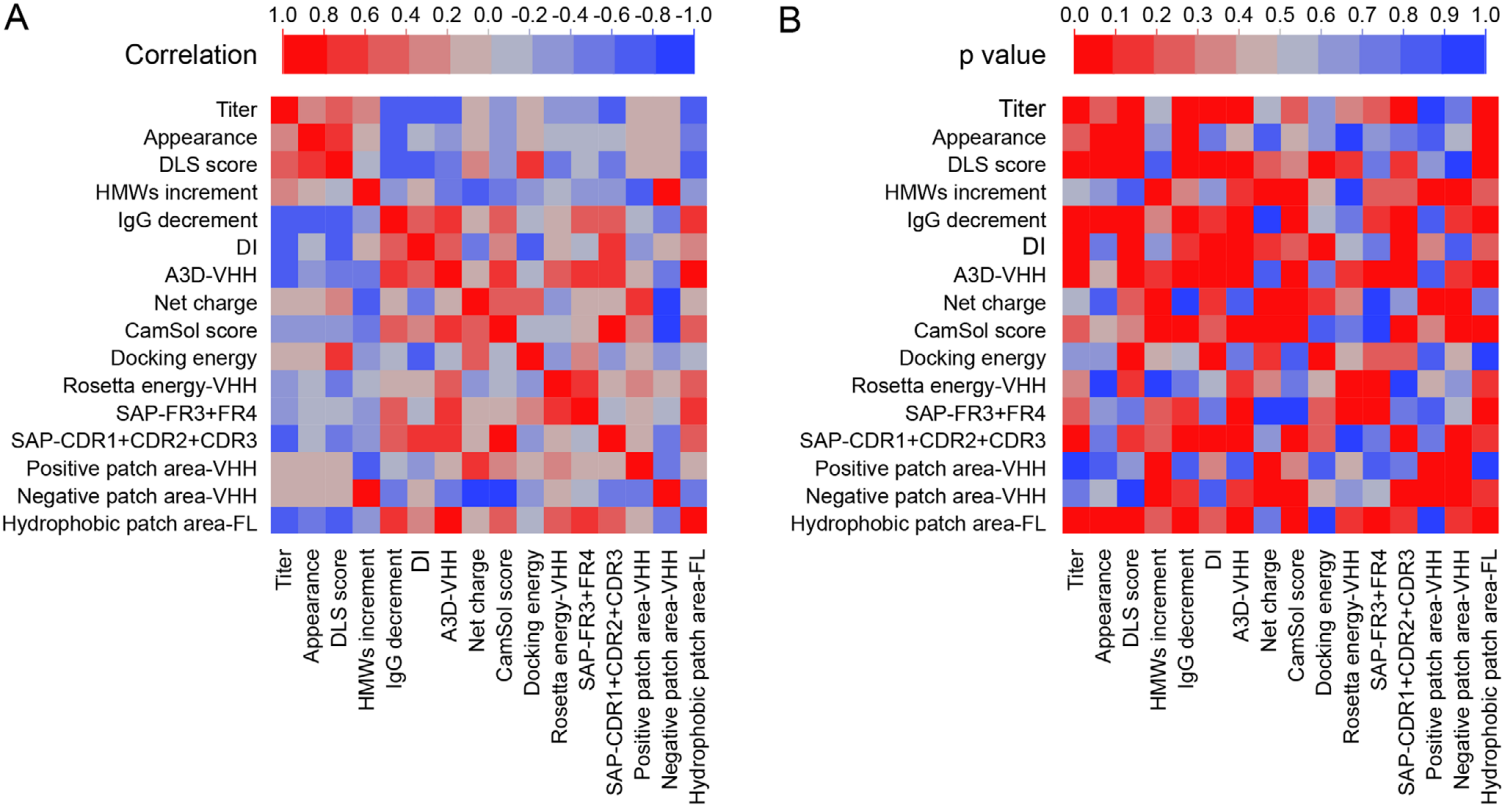
Correlation analysis between calculated structural features and experimental data. A) The heatmap of the correlation coefficient values (R). As the color bar shows, the more saturated the color, the stronger the correlation. B) The heatmap of the probability of significance (*p*). A correlation is considered significant with r > 0.5 and *p* < 0.1.

The variant design has suggested that the structural features of the antibodies obtained from the computational simulations and evaluations are closely related to their stability. To directly apply such features to predict the attributes of antibody stability, we used the JMP software (Ver.16) to analyze the relationships between the calculated structural features and the stability attributes (**Figure** **10**). In the analyses, we considered a correlation between the calculated structural features and the stability attributes as significant when the correlation coefficient r > 0.5 and the statistical significance *p* < 0.1.

As shown in Figure 10, the hydrophobic patch area is correlated with the solution turbidity and the visible particles of antibodies (r: −0.53, p: 0.080): A smaller hydrophobic area is less likely to produce the protein particles on the macroscopic length. This also suggests that the visible protein particles of VFA01 are mainly mediated by the hydrophobic interactions. And, the structural features related to the increase in the DLS average hydrodynamic diameter include: docking energy value (r: 0.64, *p*: 0.024), DI value (r: −0.63, *p*: 0.027), hydrophobic area (r: −0.63, *p*: 0.029), and A3D value of the VHH region (r: −0.53, *p*: 0.075). They indicate that the hydrophobic and electrostatic interactions co‐mediate the formation of the sub‐micrometer scale aggregates: Reducing the hydrophobic area and increasing the net charge can mitigate the sub‐micrometer aggregation. The structural features related to the increase in SEC HMW% are: negatively charged area (r: 0.81, *p*: 0.002), net charge (r: −0.74, *p*: 0.006), positively charged area (r: −0.67, *p*: 0.016) and CamSol score (r: −0.57, *p*: 0.052), indicating that aggregates below 100 nm are mainly mediated by the electrostatic interactions. As a result, increasing the protein net charge, increasing the positively charged area, and reducing the negatively charged area can decrease SEC HWM%. Stability experiments have also shown that mutation to K is beneficial in inhibiting the aggregation, while the mutation to D promotes the aggregation. Interestingly, the production yield is also related to the hydrophobic and electrostatic interactions, as: A3D value (r: −0.78, *p*: 0.003), DI value (r: −0.68, *p*: 0.016), and hydrophobic patch area (r: −0.67, *p*: 0.017). Therefore, effective control of the protein aggregation may increase the production yield.

In addition, the protein degradation is related to these features: hydrophobic area of the full length (r: 0.79, *p*: 0.002), A3D value of the VHH region (r: 0.76, *p*: 0.004), CamSol score (r: 0.59, *p*: 0.042), and negative patch area (r: −0.43, *p*: 0.163), indicating that the structural flexibility and hydrophobic and electrostatic interactions mediate the degradation. Increasing structural flexibility and a larger hydrophobic area are associated with a higher degradation propensity. In fact, the stability experiments have also confirmed that the mutation to D inhibited the protein degradation, while the mutation to K accelerated the degradation.

Based on the above statistical analyses, it is likely that VFA01 initially forms soluble oligomers through electrostatic interactions. Thus, increasing the positively charged areas and decreasing the negatively charged areas may improve its colloidal stability and thereby reduce oligomer formation. As the aggregation reinforces, the intermolecular interactions gradually shift from electrostatic to hydrophobic, resulting in aggregates from the sub‐micrometer to the micrometer scale, or even the visible particles. Docking energy, DI value, and hydrophobic area, which reflect both hydrophobic and electrostatic interactions, can better predict the tendency to aggregate at this scale. Therefore, the strategy for optimizing antibody stability should vary according to the size of the aggregates. It is also worth noting that mutating STAP to the positively charged K can reduce protein aggregation, but also results in more protein fragments. Conversely, mutating STAP to D has the opposite effect. Therefore, an effective mutation design depends on the specific stability issues of the given antibody.

## 3 Discussion and Conclusion

In this study, we have developed a computational pipeline to analyze the stability of an anticancer VHH‐Fc fusion antibody (VFA01) and design its stable variants. We evaluated its conformational stability, disulfide bond reduction state, and aggregation and degradation tendency, and then identified the hotspot residues affecting the stability: C130, F57, Y106, L120, and W111. Based on these hotspots, we designed a series of VFA01 variants and successfully obtained a variant M11 (C130S/W111F/F57K) whose stability was significantly improved compared to VFA01. In addition, the antigen‐binding activity and the production yield were also improved by approximately 1.5‐fold. Significantly, we also found that the calculated structural features of VFA01 and its variants correlated well with their protein stability properties, providing valuable guidance for antibody stability optimization.

As known, due to technological limitations, most current therapeutic antibody screening studies focus on their biological activities but usually neglect their physicochemical properties. Such an approach often leads to development problems such as low production yield, poor protein stability, and clinical failure. Although upstream and downstream processes and formulation development are used to address the stability and production yield issues, such process solutions have a long development cycle and typically reduce the production yield. It is therefore important to assess the protein stability of antibody candidates at the molecular discovery stage and then improve CMC (Chemistry, Manufacturing, and Controls) issues through rational protein design. However, to date, there have been few comprehensive studies of protein stability assessment and optimization pipelines for therapeutic antibodies such as VFA01. In this regard, our study has developed a computational pipeline that can identify the determinants and risk factors for poor protein stability through a combination of computational simulation and experimental verification. Guided by the mechanistic models of aggregation and degradation, we have used this approach to design new antibody variants that improve stability without reducing the binding activity.

Our pipeline has several advantages over traditional methods. First, by integrating homology modeling and MD simulations, this approach can predict accurate three‐dimensional structures for the computational evaluations of the antibody. Second, its computational analysis can comprehensively evaluate the conformational stability, the reduction state of disulfide bonds, and the tendencies toward aggregation and degradation, which were often neglected in previous studies or could only be roughly estimated by experimental methods. In particular, this method combined the binding mode and the binding energy to identify possible protein aggregation conformation and determined the key STAPs through the PPI preference and A3D scores. In fact, based on the findings of this pipeline, we have successfully designed the variant M11 that significantly improved the stability, binding affinity, and production yield. Therefore, the computational process and variant design strategy proposed in this study provide an efficient and accurate method to improve the stability of VHH‐Fc fusion antibodies (HcAbs).

Of course, limitations remain within our physics‐based methods. For example, our method predicted the oxidative risk sites merely by the solvent‐accessible surface area (SASA%). But, as we have shown, the risk for oxidation was not only related to the residue exposure to solvents but also potentially associated with neighboring residues. Further optimization of the prediction method for the oxidative risk sites is needed in the future. In addition, based on our understanding of peptide bond cleavage mechanisms, a more accurate predictive model for peptide bond cleavage is needed. Moreover, this study mainly focused on single‐point mutations, more complex designs by mutation combinations might be introduced for effective optimization as certain mutations can affect internal or external interaction sites, leading to long‐range effects and global structural changes.^[^ ^36^ ^]^ In recent years, artificial intelligence (AI) has demonstrated significant potential in protein stability and mutation design.^[^ ^37^, ^38^, ^39^, ^40^ ^]^ From the success of AlphaFold to the applications of various deep learning methods, AI machine learning models offer new perspectives for antibody optimization.^[^ ^41^ ^]^ In general, physics‐based modeling is ideal for providing mechanistic insights, simulating rare events, and validating predictions, but it is computationally expensive and less scalable; and machine learning models are good at handling large datasets, making rapid predictions, and discovering novel patterns, but they require high‐quality data and may lack interpretability. Specifically for antibody engineering, machine learning models still need to address challenges such as data quality, model generalization ability, and algorithm interpretability.^[^ ^42^, ^43^ ^]^ Therefore, in the future a combination of machine learning with our physics‐based approach may yield the best results, leveraging the strengths of each to overcome their respective limitations.

In summary, VHHs or nanobodies are important therapeutic antibodies and have typically been fused to the Fc fragment of full‐length antibodies to prolong their half‐life. To address their stability issues, in this study, we successfully developed an in silico pipeline to analyze the stability of an anticancer VHH‐Fc fusion antibody (VFA01) and design its stable variants. In this way, we finally obtained a variant M11 (C130S/W111F/F57K) whose stability was significantly improved compared to VFA01. Therefore, the results of our study demonstrated that our computational pipeline is a very promising approach to improve the protein stability of therapeutic VHH‐Fc fusion antibodies.

## 4 Experimental and Computational Section

### Materials

VFA01 and its variants were expressed in Chinese hamster ovary cells and produced by Henlius (Shanghai, China). The titers of VFA01 and its mutants were determined with PA‐HPLC.^[^ ^44^ ^]^ The harvests of the cell culture fluids (HCCF) were processed through depth filtration step, affinity chromatography, virus inactivation, cation exchange chromatography (CEX), and anion exchange chromatography (AEX) to obtain purified proteins. The mAbs were then ultrafiltered and diafiltered to a concentration of 20 mg mL^−1^. Then, proteins from different batches were formulated in 20 mm histidine buffer, 100 mm sucrose, and 0.02% PS20 at pH 6.0. Variants were prepared in the formulation composed of 20 mM histidine buffer, 58 mM sucrose, and 0.02% PS80 at pH 5.4, with VFA01 in the same formulation. The protein concentration was determined by measuring absorption at 280 nm with a DropSense 16 (Trinean, USA). All formulation excipients were of compendial (ChP/USP/EP) grade. The sucrose was from Merck KGaA (Darmstadt, Germany), the histidine and histidine hydrochloride were from Kyowa Amino Acid (Shanghai, China) and the polysorbate 20 was from Nanjing Well Chemical (Nanjing, China). Other chemicals were purchased from Sigma Aldrich (St. Louis, USA) without special mention.

### Accelerated Storage Stability Test

Protein long‐term stability under normal storage conditions was assessed using the standard accelerated storage stability test in which antibodies were initially stored at an elevated temperature of **4**0 °C **for 4 weeks**. The VFA01 samples in target formulations were filtered through 0.22 µm syringe filters (Merck, USA) and aliquoted into 2R vials. Sealed vials were then stored at 40 °C for 4 weeks. Samples stored at 2–8 °C were used as controls as needed.

### Antigen‐Antibody Binding Capacity Analysis

Binding activities of VFA01 and its variants were measured using an internal method not to be disclosed publicly based on the principle of ELISA. Mainly, VFA01 can bind the targeted antigen and then specifically binds HRP‐labelled IgG antibody, followed by an enzymatic reaction to produce colored enzyme product. The 50% effective concentrations (EC_50_ values) of the reference standard and the test sample are calculated using the four‐parameter logistic fit method, and EC_50_ values are used to calculate the relative antibody‐antigen binding capacity of VFA01.

### Peptide Mapping

Samples were solubilized in 6 m guanidine hydrochloride and 50 mm tris hydrochloride (Tris‐HCl) at pH 8.0, then reduced with 10 mm DTT or alkylated with 20 mm iodoacetamide (IAM). The reduced or alkylated samples were then desalted and buffer exchanged to a pH 8.0 digestion buffer with a NAP‐5 column (GE Healthcare, USA), and then digested with trypsin (Promega, USA). The resulting peptides were subsequently injected onto an ACQUITY UPLC BEH C18 column (300 Å, 1.7 µm, 2.1 × 150 mm, Waters, USA) coupled online to Thermo QE Plus system (Thermo Fisher Scientific, USA) for separation and detection. MS data were processed by Biopharma Finder software (Thermo Fisher Scientific, USA). For the enzyme digestion limitation, W111 and W115 are located on the same peptide. Thus, the oxidation percentage for W111 and W115 indicates the proportion of peptides with at least one of these tryptophan residues oxidized. No peptides were detected with both W111 and W115 oxidized simultaneously. The other amino acid residues of interest were each located on separate enzymatic peptides.

Disulfide bonds were analyzed by non‐reduced and reduced peptide mapping. Samples for non‐reduced peptide mapping were solubilized in 6 m urea and 50 mm NaAc‐HAc (pH 5.0) and alkylated with 5 mm nethylmaleimide. Next, the alkylated or reduced protein samples were buffer exchanged into a pH 8.0 digestion buffer using centrifugal filter, and then digested by LysC and trypsin at 37 °C overnight. Then, half of the enzymatic digests were reduced with DTT, and the other half alkylated with IAM. Both the resulting disulfide bond linked peptides and reduced peptides were identified by LC‐MS/MS analysis as mentioned above.

### Intact and Reduced Molecular Weight Analysis

The molecular weights of intact and reduced VFA01 were determined by an ACQUITY UPLC BEH C4 column (300 Å, 1.7 µm, 2.1 × 50 mm, Waters, USA) coupled online to Agilent 1290 UHPLC‐6545XT Q‐TOF mass spectrometer (Agilent, USA). The samples used for reduced analysis were treated with dithiothreitol (DTT) for reduction. 0.5 µg of each sample was injected and data in the m/z range of 400–6000 were acquired. LC‐MS data were processed by Agilent BioConfirm software (Agilent, USA).

### Differential Scanning Fluorimetry (DSF)

Differential Scanning Fluorimetry (DSF) was performed on an UNcle system (Unchained Labs, USA).^[^ ^45^ ^]^ Intrinsic fluorescence intensity from tryptophan was monitored. 9 µL sample with concentration at ≈20 mg mL^−1^ were loaded in the Uni sample loader (Cat. No. 201–1009). The temperature was set to increase from 25 to 95 °C with 1 °C min^−1^ as ramp rate. The excitation wavelength was set to 266 nm, and the emission spectrum was collected from 266 to 473 nm. The data analysis was done using the UNcle Analysis software to obtain the onset melting temperature (*T*_m_onset_), defined as the temperature at which the first derivative curve begins to rise in the barycentric mean (BCM) curve of fluorescence emission peaks.

### Visual Inspection

Visual inspection was used to observe visible particles and turbidity of the samples. Prior to turbidity/chromaticity measurements, the sample vials were gently inverted 10 times to ensure sediment resuspension. Then, the vials were observed at 3750 lux according to Ph.Eur. Chapter 2.9.20. The turbidity and visible particles of each vial were observed for at least 5 s in front of a black background while the color was observed against a white background. The appearance of particles and the change in turbidity and color were recorded.

### Subvisible Particle Analysis

The FlowCam 8100 (Fluid Imaging Technologies, USA) was used for detection of the sub‐visible particles. A 250 µL aliquote was analyzed with 10 × magnification at 0.1 mL min^−1^ flow rate and 18 frames per second AutoImage frame rate. A segmentation threshold of 15.0 for dark and light pixels was applied. After each measurement, the flow cell was washed with 1% Tergazyme and rinsed with water pre‐treated with 0.22 µm filter. Sub‐visible particles bigger than or equal to 1, 2, 5, 10, and 25 µm were evaluated with Visual Spread sheet software Version 4.7.6 (Fluid Imaging Technologies, USA) and are presented as counts per mL.

### Dynamic Light Scattering (DLS) Analysis

DLS analysis was conducted using a DynaPro Plate Reader II (Wyatt Technologies, Germany). Each well was analyzed with 10 acquisitions and an acquisition time of 5 s. The detection results were analyzed with DynaPro Dynamics 7.10 software (Wyatt Technologies, Germany). After adding 25 µL sample solution into a 384‐well plate, the plate was centrifuged at 2000 rpm for 2 min to remove trapped air from the plate bottom. The samples were then detected by isothermal mode at 25 °C.

### Size‐Exclusion Chromatography (SEC)

SEC was performed on an Agilent 1200 series HPLC system. 50 µg per sample were injected onto a TSKgel G3000SWXL HPLC column (7.8 × 300 mm, 5 µm, Tosoh Bioscience, Germany) using a mobile phase of 100 mm sodium dihydrogen phosphate pH 6.8 with 150 mm sodium chloride at a flow rate of 0.5 mL min^−1^. Chromatograms were analyzed with ChemStation software (Version B.02.01‐SR2, Agilent, USA) regarding retention time and area under the curve (AUC) after UV detection at 280 nm.

### Capillary Electrophoresis Sodium Dodecyl Sulfate (CE‐SDS)

CE‐SDS was detected with the Beckman PA800(s) (Beckman Coulter, USA), using detection at 220 nm. Entangled polymer solution is introduced into a capillary, where it serves as the sieving matrix, separating proteins by size as they migrate through the gel. The method is used to measure product purity and detect any new impurities that appear during stress treatment. The samples were first diluted with water to 4 mg mL^−1^. Further preparation of the reduced samples included adding 2 µL 10 kDa Internal Standard, 7 µL reduction agent β‐ME, 75 µL SDS sample buffer (100 mmol L^−1^ Tris‐HCl, pH 9.0, 1% SDS), and water into the diluted sample to a final volume of 109 µL, then incubating the reduced samples at 80 °C for 10 min. Preparation of the non‐reduced samples was almost the same as the reduced samples except for 7 µL 0.25 m IAM solution was added instead of β‐ME.

The separation cartridge used contained a 50 µm × 20 cm bare fused silica capillary. The sample injections were conducted electrokinetically with −5 kV for 20 s, while separation was conducted with −15 kV for 35 min. The capillary was kept at 8 °C during the whole procedure.

Detection was done UV‐metrically at a wavelength of 220 nm. Data were analyzed using 32 Karat Software (Beckman Coulter, USA).

### Charge Variants Detection

Cation Exchange Chromatography (CEX) was performed on a Thermo Scientific ProPacTM WCX‐10 BioLC column (4 mm × 250 mm, packed with 5 µm pellicular particles) attached to an Agilent HPLC 1260 using a mobile phase A (referred to as A in this part) comprising 50 mm phosphate buffer (pH 6.1) and a mobile phase B (referred to as B in this part) comprising 50 mm phosphate buffer and 300 mm NaCl (pH 6.1). An ionic strength gradient was started with equilibration of the column using 4% mobile‐phase B for 3 min followed by 4–31% mobile‐phase B for 28 min. Then a steep increase to 100%B within 1 min was applied and the column was flushed with 100%B for 2 min and re‐equilibrated with 4%B for 5 min. Other parameters were set as follows: flow rate 1 mL min^−1^, temperature 35 °C, and UV detection at 280 nm.

### Imaging Capillary Isoelectric Focusing (icIEF)

icIEF is the imaged capillary version of the IEF technique for separating proteins by differences in their isoelectric points (pI). Samples were diluted to 1 mg mL^−1^ and premixed with 140 µL premixing solution (Pharmalyte (8–10.5), 2 µL pI Marker (pI 7.65 and pI 10.10), 10 m urea solution, and 1.0% methyl cellulose (MC)) to a final volume of 200 µL. 100–180 µL of the above solution was transferred to the sample vial for the injection. The experiments were performed using iCE3 Protein Simple (Bio‐Techne, USA). The sample injection duration was set as 60 s at 10 °C. Focusing was carried out with 1500 V for 1 min and 3000 V for 11 min. Data analysis was performed by labeling the pI 7.65 and pI 10.10 and calculating the pI of the main peak by iCE CFR software. The main peak, the acidic and basic peaks were separated.

### Molecular Dynamics Simulation

The 3D structures of the VHH domain and the conserved Fc domain of VFA01 were predicted by homology modeling via SWISS‐MODEL, and the hinge region was constructed by MODELLER (Ver.10.2) based on the template structure 1IGT (PDB ID).^[^ ^46^ ^]^ Then, the initial structures of VFA01‐2SS and ‐3SS were assembled directly with PyMOL based on the structure of VFA01. Note that we once used AlphaFold2/AlphaFold3 to predict the entire structures of VFA01 and its variants using their sequences as input. However, the predicted structures were not correct because two VHH domains were predicted to be in very close contact with the Fc domain. Therefore, we used the 3D models predicted by homology modeling in this study.

MD simulations of the VFA01 systems were performed using the GROMACS (Ver.2022) package,^[^ ^47^ ^]^ with the CHARMM36 force field used for system parameters.^[^ ^48^ ^]^ For each system, VFA01 was placed in the center of a rectangular box, with a minimum distance of 10 Å between its surface and the box edge. The simulation system was solvated using the TIP3P water model, and Na^+^ and Cl^−^ ions were randomly added to neutralize the system and achieve an ionic concentration of 150 mm. Simulations were performed with the periodic boundary conditions. The long‐range electrostatic interactions were calculated by the Particle‐Mesh‐Ewald (PME) method,^[^ ^49^ ^]^ and the cut‐off distances of the short‐range electrostatic and van der Waals interactions were set to 14 Å. The system was then energy‐minimized using the steepest descent method for 5000 steps, followed by a 50 and 100 ps position‐restricted MD simulation under *NVT* and *NPT* ensembles, respectively. Finally, the system underwent a 500 ns simulation with all constraints removed. All bonds were constrained using the LINCS algorithm,^[^ ^50^ ^]^ and the simulation time step was 2.0 fs. The pressure of the *NPT* ensemble was set to 1 atm and controlled by the Berendsen algorithm,^[^ ^51^ ^]^ and the temperature was set to 300 K and maintained by the Velocity rescaling algorithm.^[^ ^52^ ^]^ The production simulations were performed in the *NPT* ensemble for at least 500 ns. The MD trajectories were analyzed for the calculations of RMSD, Rg, RMSF, and SASA by corresponding modules in GROMACS.

### Free Energy Calculation by Non‐Equilibrium Simulation

Crooks Gaussian Intersection (CGI) method through non‐equilibrium MD simulation trajectories was used to calculate the free energy difference (Δ*G*) between reduced and oxidized states in the redox reaction, which is the intersection point of the forward (from oxidized to reduced state) and backward (from reduced to oxidized state) work distributions:^[^ ^21^, ^53^ ^]^

(1) $$\frac{P_{f}\left(W\right)}{P_{b}\left(-W\right)}=\exp\left[\beta \left(W-\Delta G\right)\right]$$

where *P*_f_(*W*) and *P*_b_(‐*W*) are the forward and backward work distributions.

To estimate the free energy difference Δ*G*, a non‐equilibrium transition process was constructed based on the methods in previous studies.^[^ ^21^, ^24^ ^]^ In the oxidized state, the disulfide bond was formed by two Cys residues. And in the reduced state, two normal Cys residues are present. For the end state of oxidation, the 200 snapshots between 480–500 ns were extracted from the production simulations of 3SS. For the end state of reduction, 50 ps *NVT* equilibrium simulations are carried out, followed by 50 ns *NPT* production simulations. Subsequently, 100 snapshots between 40–50 ns were extracted every 100 ps. Finally, 2 ns × 300 replicas of non‐equilibrium simulations (backward and forward directions, Δλ = 0.4 fs) were performed to alchemically morph between the oxidized and reduced states. The work values derived from the simulations were used to estimate Δ*G* by the pmx program. The redox potential of three disulfide bonds (C130─C130, C136─C136, and C139─C139) in the hinge regions was calculated to predict their reduced tendency.

### Redox Potential Calculation

The redox potential (*E* ^0^) was calculated based on the Nernst equation:

(2) $$E^{0}=-\Delta G/nF$$

where Δ*G* is the free energy difference between the reduced and oxidized states in a redox reaction and was calculated by the CGI method above, *n* is the number of electrons transferred, and *F* is the Faraday constant.

### Property Calculations on 3D Models

Surface property patches, consisting of both hydrophobic and ionic patches, were identified with the protein properties applications in MOE 2022.^[^ ^54^ ^]^ Hydrophobic patches were considered if they exceeded 50 Å^2^ as determined by mapping atomic SLogP onto the protein surface. Ionic patches were formed when there was sustained excess of the force field charge (Amber10:EHT) over a surface area of 40 Å^2^. The pI and net charge are calculated using the PROPKA method to determine residue pKa values.^[^ ^55^ ^]^ The CamSol algorithm was employed for predicting aggregation, incorporating three biophysical properties: hydrophobicity, charge, and propensity to adopt α‐helical and β‐sheet secondary structures.^[^ ^56^ ^]^ The internal molecular energy of VFA01 (or a variant) was calculated using the *score_jd2* module in Rosetta, which utilizes the score function for calculating the energy of all atomic interactions within a protein.^[^ ^46^ ^]^

### Protein─Protein Docking

The global dockings of VFA01 were performed with Cluspro (https://cluspro.org/) to obtain the possible conformation of aggregation.^[^ ^28^ ^]^ The docking method was direct docking. There are two possible structures for VFA01, 2SS, and 3SS. Therefore, there are three possible docking combinations: 2SS–2SS, 2SS–3SS, and 3SS–3SS. In the docking combination 2SS–2SS, both the receptor and ligand proteins are 2SS. In combination with 2SS–3SS, the receptor protein is 2SS and the ligand protein is 3SS. In combination with 3SS‐3SS, both the receptor and ligand proteins are 3SS. Subsequently, all models for all energy coefficients were downloaded as the possible aggregation conformations for VFA01.

The global docking of 2SS and the antigen TIGIT was performed using Cluspro to obtain the possible conformation of VFA01 binding to the antigen. The direct docking was performed with 2SS referred to as the receptor and TIGIT was chosen to be the ligand. Subsequently, all energy coefficient models were downloaded, and the conformation of VHH binding to TIGIT was screened out as the possible binding mode.

### Protein–Protein Interface Energy Calculation

To evaluate the energy at the interface of the docking conformations, the *interface_energy* module of Rosetta was used to calculate the pairwise energies of all interfacial residue pairs between the receptor and ligand proteins.^[^ ^57^, ^58^ ^]^ Each pairwise energy includes short‐range, context‐dependent long‐range, and context‐independent long‐range terms as defined in Rosetta. The total protein‐protein interface energy is the sum of the pairwise energies of all interfacial residue pairs.

### PPI Preference Calculation

To calculate the PPI preference of each residue for each docking conformation, the contacts between the receptor and the ligand atoms within a 5 Å cutoff were counted and summed over poses.^[^ ^57^ ^]^ The final PPI preference value was assigned by normalizing the number of contacts per residue by the maximum number of contacts.

### AGGRESCAN3D (A3D) Calculation

To predict the structural aggregation‐prone regions (STAP) on the aggregation interface, A3D was used to comprehensively score the VHH structure of the single‐domain heavy chain antibody, and calculate the A3D value of each residue.^[^ ^27^ ^]^ The A3D value mainly characterizes the hydrophobicity of each residue and thus the induced aggregation tendency. The A3D score of 1.0 was then set as the threshold to identify the sites with potential aggregation tendencies.

### Alanine Scan

To identify the major binding residues between VFA01 and its antigen, alanine scanning was performed with MOE after obtaining their complex structures.^[^ ^59^ ^]^ First, amino acid residues located at the binding interface were identified through the complex structure, and then these residues were mutated to alanine one by one. Then, the conformational optimization of the mutant complex was performed using the LowMode method, and the affinity before and after mutation was calculated. From the difference in affinity, the key amino acids that affect binding were identified. A positive difference indicated that the alanine mutation at this site was not favorable for the stability of the complex, and a negative difference indicated that the alanine mutation at this site favored the stability of the complex.

### Developability Index (DI)

To evaluate the effects of hydrophobic and electrostatic effects on aggregation, we used *DI* to characterize:^[^ ^60^ ^]^

(3) $$DI=\left(HcAb\;SAP\;score\right)\;-\beta \times {\left(HcAb\;net\;charge\right)}^{2}$$

where β is the regressed parameter (0.0498).

### Spatial Aggregation Propensity (SAP)

The SAP tool was developed to identify the hydrophobic region on the protein surface.^[^ ^61^ ^]^ SAP has been applied to predict aggregation‐prone regions of proteins, including monoclonal antibodies. SAP is defined as:

(4) $$SAP_{atom\;i}=\sum_{\begin{array}{c} simulation\;\\ average \end{array}}\left\{\sum_{\begin{array}{c} Residue\;with\;at\;least\;one\\ atom\;within\;5\;Å\;of\;atom\;i \end{array}}\left[\frac{SAA\;of\;side\;chain\;atoms\;within\;5\;Å}{SAA\;of\;side\;chain\;atoms\;of\;fully\;exposed\;residue}\times Residue\;hydrophobicity\right]\right\}\;$$

The SAP score of a protein was determined by the sum of all positive SAP values in its complementary determination region (CDR). The definition of the CDR used in this work is the definition used by the KABAT homology algorithm.

(5) $$SAP\;score=\sum_{\begin{array}{c} All\;atoms\;in\;CDR\;\\ with\;SAP\;value>0 \end{array}}\left(SAP_{atom\;i}\right)$$

The net charge of a HcAb was calculated using ProPKa3.0,^[^ ^62^ ^]^ with the full structure.

### Correlation Analysis

The correlation between experimental test data and calculated structural features was analyzed by the multivariate methods module of JMP software (Ver.16). The experimental data was further processed, as the appearance of the samples during the stability test was graded into 5 levels (1, 3, 5, 7, and 9). Higher score corresponds to a better appearance, which means fewer and smaller visible particles appeared in the samples. The DLS score used the same scoring system as appearance. The higher score indicates fewer particles with larger diameters appeared. The HMWs increment, as well as the antibody (Ab) increment, was calculated using the formula: (HMWs%/intact Ab% at Day 28 – HMWs%/intact Ab% at Day 0) ÷ 4 weeks. The two factors with r >0.5 and *p* < 0.1 are considered significantly correlated.

### Statistical Analysis

Microsoft Excel and JMP software (Ver.16) were used to perform statistical analyses in this study. Correlations were analyzed using Pearson's correlation. Data were expressed as mean ± SD. A paired sample *t*‐test was used to compare differences between groups. The difference was defined as statistically significant with *p < 0.05, **p < 0.01, and *** *p <* 0.001 in the figures and tables where applicable.

## Conflict of Interest

The authors declare no conflict of interest.

## Supporting information



Supporting Information

## Data Availability

The data that support the findings of this study are available from the corresponding author upon reasonable request.
