# Supplementary material for: Enhancing the Protein Stability of an Anticancer VHH‐Fc Heavy Chain Antibody through Computational Modeling and Variant Design
Source: Adv Sci (Weinh). 2025 Apr 24;12(23):2500004. doi: 10.1002/advs.202500004 (PMC12199382; doi:10.1002/advs.202500004)
Supplement: Supplementary file 1 — Supporting Information [file ADVS-12-2500004-s001.pdf]

ADVANCED SCIENCE
Open Access

Supporting Information

for *Adv. Sci.*, DOI 10.1002/advs.202500004

Enhancing the Protein Stability of an Anticancer VHH-Fc Heavy Chain Antibody through Computational Modeling and Variant Design

Yuan Fang, Menghua Song, Tianning Pu, Xiaoqing Song, Kailu Xu, Pengcheng Shen, Ting Cao, Yiman Zhao, Simon Hsu, Dongmei Han and Qiang Huang**

Supporting Information

Enhancing the Protein Stability of an Anticancer VHH-Fc Heavy Chain Antibody through Computational Modeling and Variant Design

Yuan Fang[1,2], Menghua Song[2], Tianning Pu[2], Xiaoqing Song[2], Kailu Xu[2], Pengcheng Shen[2], Ting Cao[2], Yiman Zhao[2], Simon Hsu[2], Dongmei Han[2]*, Qiang Huang[1,3]*

[1]*State Key Laboratory of Genetics and Development of Complex Phenotypes, Shanghai Engineering Research Center of Industrial Microorganisms, MOE Engineering Research Center of Gene Technology, School of Life Sciences, Fudan University, Shanghai 200438, China*

[2]*Department of Technical Operations, Shanghai Henlius Biotech, Inc., Shanghai 200233, China;*

[3]*Multiscale Research Institute of Complex Systems, Fudan University, Shanghai 201203, China*

*Corresponding authors at: School of Life Sciences, Fudan University, Shanghai 200438, China; E-mail: huangqiang@fudan.edu.cn (QH). Department of Technical Operations, Shanghai Henlius Biotech, Inc., Shanghai 200233, China; E-mail: dongmei_han1@henlius.com (DH)

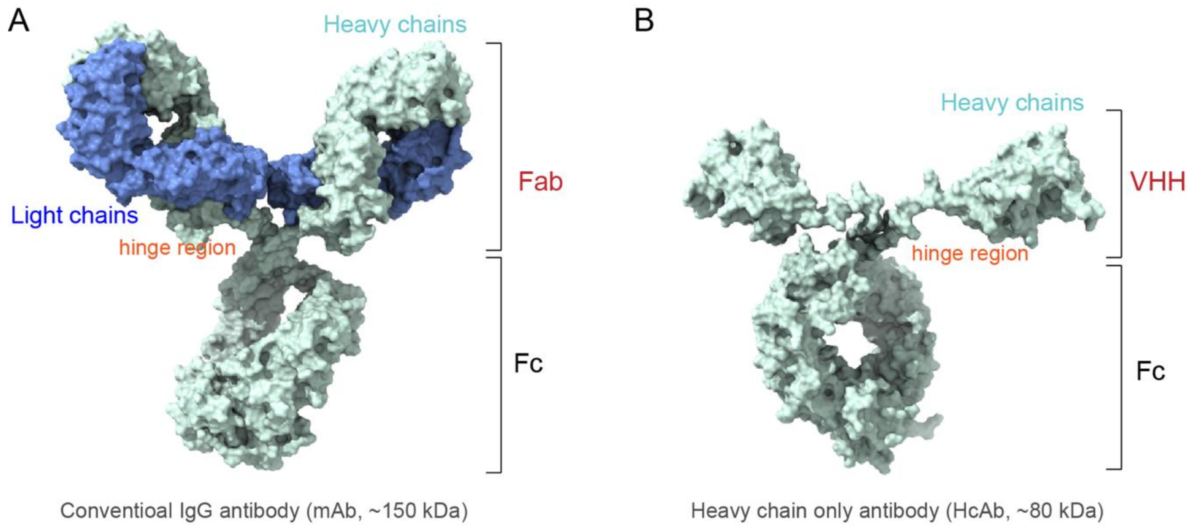

Figure S1. 3D structures of antibodies. A) A full-length IgG antibody. B) A VHH-Fc fusion heavy chain antibody (HcAb). Compared to the full-length IgG antibody, the HcAb does not have two light chains.

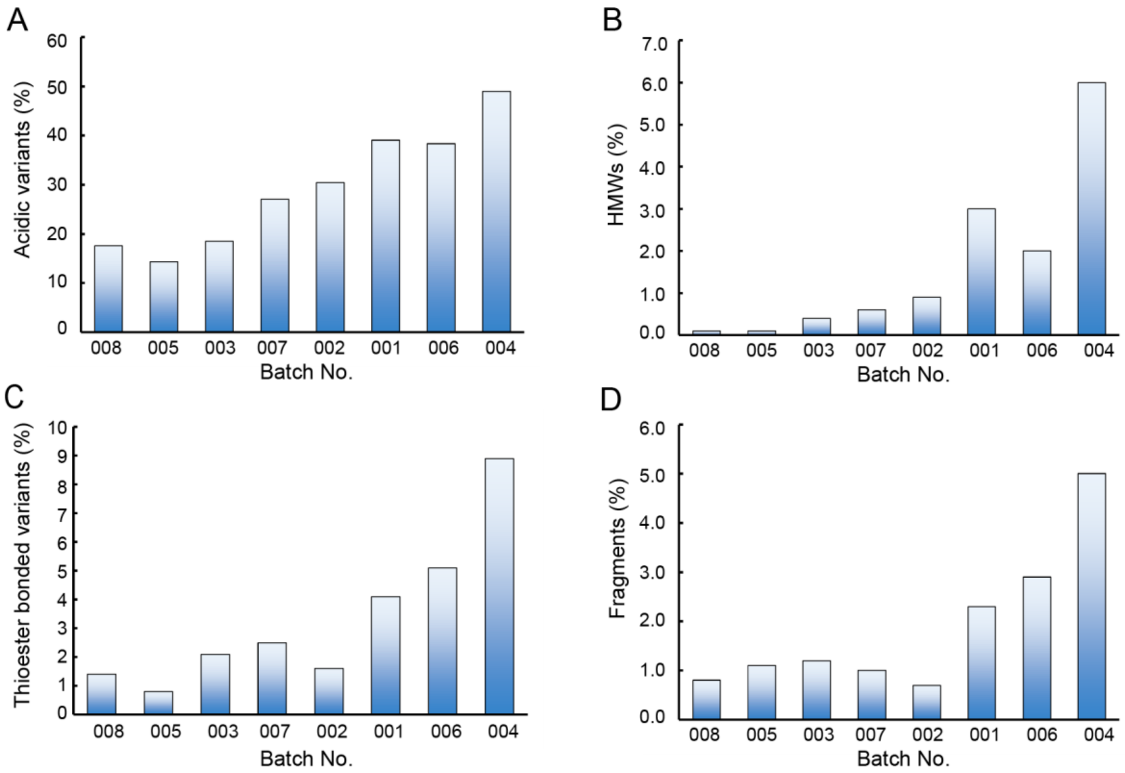

Figure S2. Quality data of the HcAb VFA01 from batches 001~008 of 5 L production after purification and formulation. A) Acidic variants detected by iCIEF. B) High molecular weight (HMW) species detected by SEC. C) Thioester-bonded variants detected by reduced CE-SDS. D) Fragments detected by non-reduced CE-SDS. Batches are ranked in order of stability from best to worst.

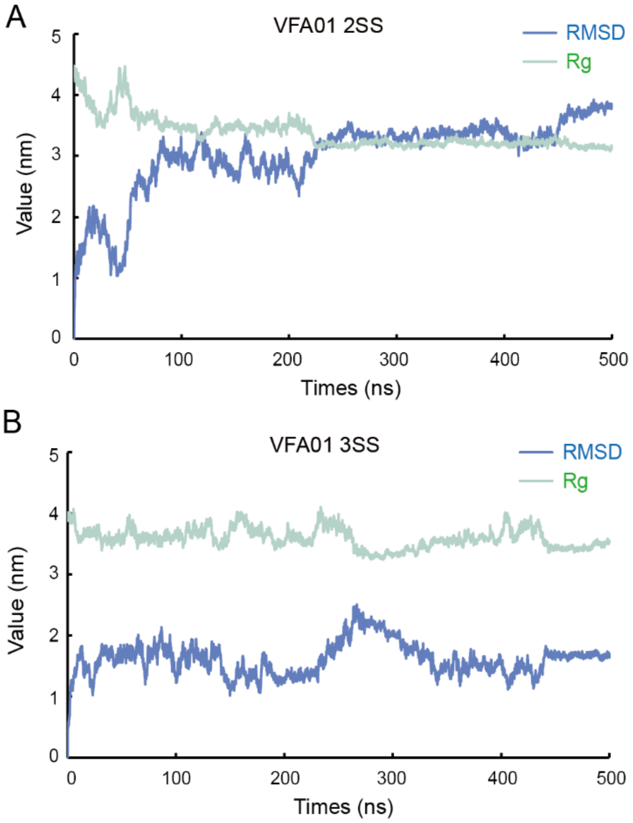

Figure S3. Time-dependent RMSD (root mean square deviations) and Rg (radius of gyration) values of VFA01 2SS and 3SS in the MD simulations. A) VFA01 2SS. B) VFA01 3SS.

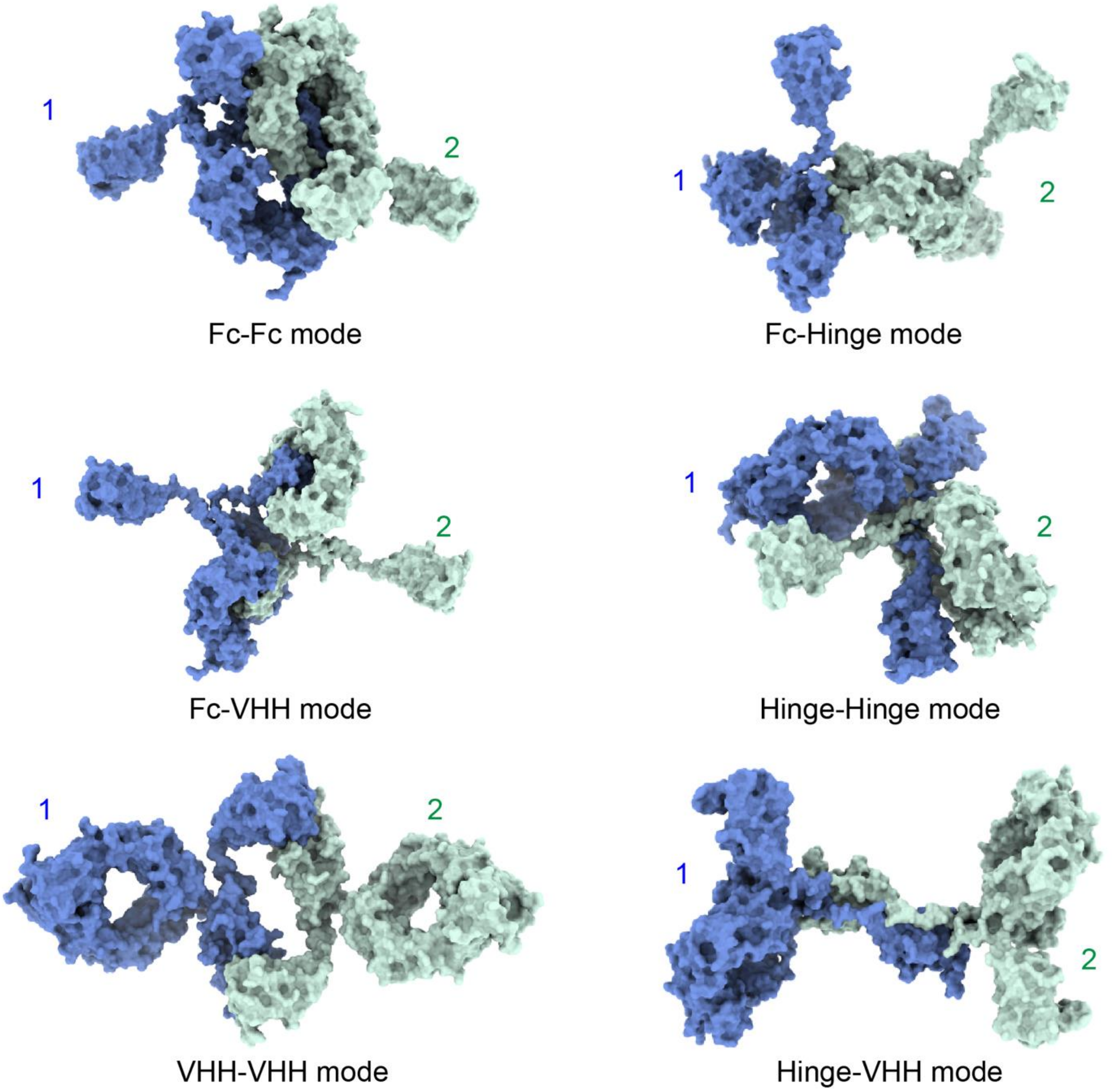

Figure S4. Six representative binding modes of two VFA01 proteins (1 and 2) in the protein-protein docking simulations. One VFA01 is shown in blue and the other one in green. The binding regions of the two proteins are indicated by the labels.

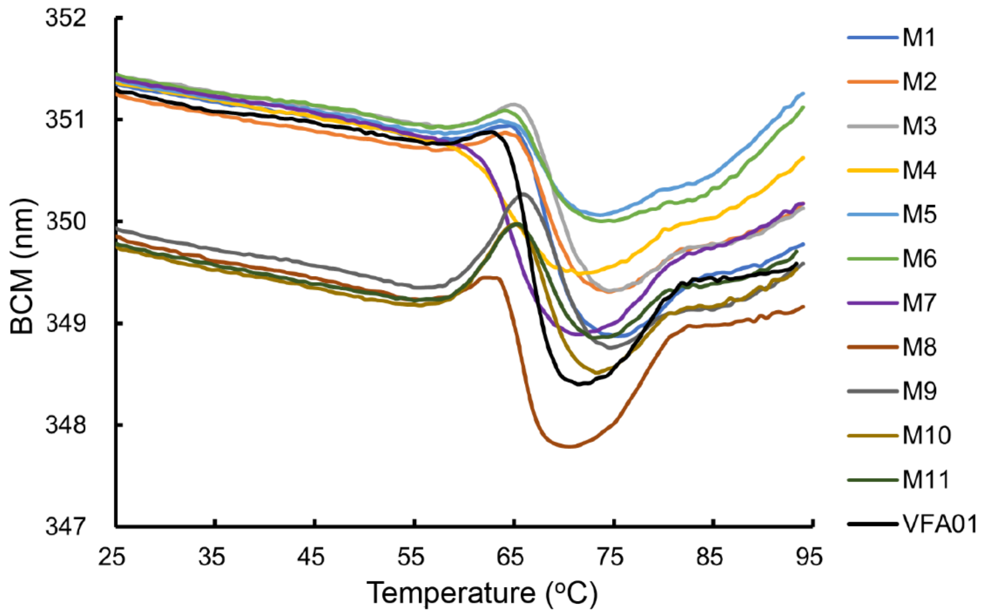

Figure S5. The DSF profiles of the HcAb VFA01 and the designed variants.

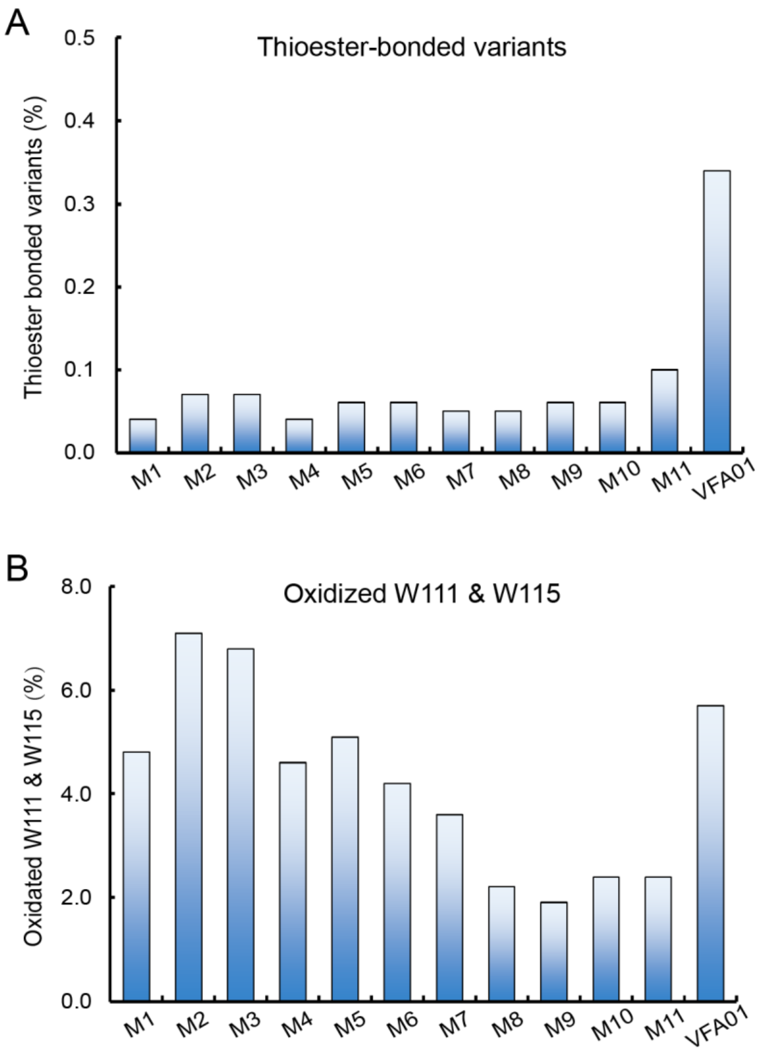

Figure S6. The percentages of the thioester-bonded variants and the oxidized W111 and W115 in the HcAb VFA01 and the designed variants at the initial time (Day 0). A) The thioester-bonded variants. B) The oxidized W111 and W115.

Table S1. Covalent linkage states of cysteine residues in the monomeric (mono.) or the aggregated (aggd.) forms of VFA01 detected by LC-MS, where the amount of each state was semi-quantified.

Residue	Form	Free thiol	Theoretical linkage	Mismatched linkage					
		C22	1:C22/1:C96	1:C22/1:C171	1:C22/1:C277	1:C22/1:C231	1:C22/1:C335	1:C22/1:C130	1:C22/1:C22
C22	mono.	2.9%	78.6%	7.8%	1.2%	2.6%	1.6%	4.5%	0.8%
	aggd.	9.8%	71.1%	10.4%	3.2%	2.3%	1.5%	1.3%	0.3%
		C96	1:C96/1:C22	1:C96/C171	1:C96/1:C130	1:C96/1:C231	1:C96/1:C96	1:C96/1:C335	1:C96/1:C277
C96	mono.	3.6%	83.1%	2.6%	3.6%	2.3%	1.2%	2.1%	1.5%
	aggd.	9.6%	72.0%	3.6%	1.7%	2.4%	2.4%	4.4%	3.9%
		C130	[a]N.A.	1:C22/1:C130	1:C96/1:C130	1:C130/1:C277	1:C130/1:C335	1:C130/1:C130	1:C130/1:C171
C130	mono.	6.3%	N.A.	27.4%	21.3%	6.5%	5.2%	0.8%	32.5%
	aggd.	11.3%	N.A.	12.4%	16.5%	20.3%	6.7%	[b]N.D.	32.9%
		C171	1:C171/1:C231	1:C22/1:C171	1:C130/1:C171	1:C96/1:C171	1:C171/1:C335	1:C171/1:C277	1:C171/1:C171
C171	mono.	4.9%	88.1%	3.1%	2.1%	1.0%	0.4%	0.2%	0.3%
	aggd.	7.4%	86.4%	2.9%	0.9%	1.0%	0.7%	0.2%	0.5%
		C231	1:C171/1:C231	1:C231/1:C277	1:C231/1:C335	1:C96/1:C231	1:C22/1:C231	N.A.	N.A.
C231	mono.	0%	96.7%	0.6%	0.6%	1.0%	1.1%	N.A.	N.A.
	aggd.	0.1%	95.4%	2.0%	1.0%	0.7%	0.7%		
		C277	1:C277/1:C335	1:C22/1:C277	1:C231/1:C277	1:C96/1:C277	1:C171/1:C277	1:C130/1:C277	N.A.
C277	mono.	1.1%	93.9%	1.1%	1.2%	1.3%	0.5%	1.0%	N.A.
	aggd.	14.3%	69.6%	3.1%	6.2%	3.7%	1.2%	2.0%	
		C335	1:C277/1:C335	1:C231/1:C335	1:C96/1:C335	1:C171/1:C335	1:C22/1:C335	1:C130/1:C335	N.A.
C335	mono.	2.3%	91.6%	1.2%	1.8%	0.9%	1.5%	0.8%	N.A.
	aggd.	24.8%	64.3%	2.9%	3.9%	2.1%	1.4%	0.6%	

[a]N.A. - not applicable; [b]N.D. - not detected.
